# How do foundation year and internship experience shape doctors’ career intentions and decisions? A meta-ethnography

**DOI:** 10.1080/0142159X.2022.2106839

**Published:** 2022-08-09

**Authors:** Yingxi Zhao, Daniel Mbuthia, Claire Blacklock, David Gathara, Catia Nicodemo, Sassy Molyneux, Mike English

**Affiliations:** Oxford Centre for Global Health Research; KEMRI-Wellcome Trust Research Programme; Oxford Centre for Global Health Research; London School of Hygiene and Tropical Medicine; Nuffield Department of Primary Health Sciences, University of Oxford; KEMRI-Wellcome Trust Research Programme in Kenya; Oxford Centre for Global Health Research

**Keywords:** Foundation year, intern, experience, career decision, specialty choice

## Abstract

**Purpose:**

Foundation years or internships are an important period for junior doctors to apply their knowledge and gain clinical competency. Experiences gained during the foundation years or internships are likely to inform newly qualified doctors’ opinions about how they want to continue their career. We aimed to understand how medical doctors’ internship experiences influence their career intention/decision.

**Methods:**

We conducted qualitative evidence synthesis using meta-ethnography. We searched six electronic bibliographic databases for papers published between 2000-2020 and included papers exploring how foundation years or internship experiences shape doctors’ career intention/decisions, including in relation to migration, public/private/dual practice preference, rural/urban preference, and specialty choice. We used the GRADE-CERQual framework to rate confidence in review findings.

**Results:**

We examined 23 papers out of 6,085 citations screened. We abstracted three high-level inter-related themes across 14 conceptual categories: (1) Deciding the personal best fit both clinically and in general (which option is “more me”?) through hands-on and real-life experiences (2) Exploring, experiencing and witnessing workplace norms; and (3) Worryingabout the future in terms of job market policies, future training and professional development opportunities. Confidence in findings varied but was rated high in 8 conceptual categories.

**Conclusions:**

Our meta-ethnographic review revealed a range of ways in which internship experience shapes medical doctors’ career intentions/decisions allowing us to produce a broad conceptual model of this phenomenon. The results highlight the importance of ensuring sufficient, positive and inspiring clinical exposure, improving workplace environment, relationship and culture, refraining from undermining specific specialities and communicating contractual and job market policies early on to young doctors, in order to attract doctors to less popular specialties or work locations where they are most needed. We propose our conceptual model should be further tested in new research across a range of contexts.

## Introduction

The foundation years (FY) or internship is an important period where doctors in training transit from supervised student learning to professional employment, and rapidly assume clinical responsibility under supervision. From this perspective internship occurs after primary academic qualification and typically includes clinical rotations through key specialties in accredited hospital settings. In most countries this is a one-year programme, though in the UK doctors undertake a two-year foundation programme. In some countries like the US, the internship is merged with specialty residency training after medical school, and the first year of residency is typically called internship (Zhao et al. 2021). Despite the different terminology and different length and context, these junior doctors experience demanding working hours and a need for ongoing learning and assessment (Essa 2011; Sturman et al. 2017; Carlsson et al. 2022) - hereafter we refer to all these positions as internships.

Internship years appear to be an important time in career decision making as this is closer to when most apply for specialty training programme or their first job (Scott et al. 2013). Experiences gained during the internship are likely to inform newly qualified doctors’ opinions about how they want to continue their career, including in which specialty, organization or even country (Scott et al. 2013). In the UK, the proportion of foundation doctors intending to pursue specialty training immediately after FY2 (the 2nd year of the foundation programme) declined from 71.3% in 2011 to 35.5% in 2019. Instead, many took up service roles in the NHS prior to committing to further training, or chose to take a break (GMC 2019). Aside from not being accepted by their preferred specialty or location, common reasons for doctors taking a break were the need to prevent or recover from burnout, uncertainty about career direction, feeling pressured to commit to a long training programme, and dissatisfaction with training environments(GMC 2019). Importantly, 11% of FY2 trainees expressed an intention to work outside of the UK temporarily or permanently, 7% intended to take a career break and 1% to leave medicine permanently (GMC 2019). Such delays or exits can have a negative impact on service delivery and workforce planning (Scanlan et al. 2019). These challenges are not unique to the UK (Cronin et al. 2019), and for low- and middle-income countries (LMICs) there are also substantial exits from public health systems and migration to high-income countries (Deressa and Azazh 2012; George and Reardon 2013; Herfs 2014; World Health Organization 2020).

Previous systematic reviews and evidence syntheses have summarised a broad list of factors that influence medical doctors’ career intentions and decision-making in relation to specialty choice (Scott et al. 2013; Puertas et al. 2013; Peel et al. 2018), retention in the public sector (El Koussa et al. 2016), in rural and under-developed areas (Mohammadiaghdam et al. 2020; World Health Organization 2021) and migration (Blacklock et al. 2014). While these reviews provide an overview of factors influencing career decisions across a range of contexts, there has been no in-depth investigation of how experiences during internship influence doctors’ ongoing career decisions. The closest is a review by Stagg et al. (2012) which suggested that positive role models during clinical rotations could change students’ career choices towards rural practice and primary care, whereas negative influences such as surgical supervisors’ disrespect to colleagues and poor teaching could “turn off” students from pursuing a surgical career (Stagg et al. 2012). Understanding the link between internship experience and ongoing career decisions is important for workforce planning, as this is a key time point to retain and direct the workforce where it is most needed. The internship period is also arguably amenable to change through action by health facilities, medical regulators, educators and policymakers.

In this review, we aimed to understand how this critical period of internship training shaped junior doctors’ career decision-making. The findings can inform medical regulators and educators, hospital administrators, and policymakers on factors to consider in strengthening internship training and career advice, especially when trying to attract junior doctors to less popular specialties and work locations.

## Materials and methods

There are different methods for qualitative evidence synthesis. We chose meta-ethnography, a theory-generating, interpretive methodology for qualitative evidence synthesis as we hoped to develop conceptual understanding through a process of constant comparison of concepts and metaphors, rather than to simply aggregate findings (Noblit and Hare 1988; France et al. 2019). We followed the 7-stage meta-ethnography approach developed by Noblit and Hare for the synthesis process (Noblit and Hare 1988). The stage-by-stage documentation of our synthesis is provided in Appendix 1.

### Search strategy and screening

With assistance from a specialist medical librarian, we conducted a systematic search using MEDLINE, Embase, Global Health, PsycINFO, Science Citation Index Expanded and Social Sciences Citation Index to identify relevant articles. We combined terms and phrases related to foundation doctors, interns, junior doctors, career choices (such as general terms, specialty choices, migration, rural, public or private sector) and qualitative studies filters (Recherche qualitative [Biblio3S]). We included papers published between 2000 and 2020 in English, and for mixed-methods studies focused only on the qualitative components. The search strategy is provided in Appendix 1.

We adopted a broad definition of “career intention and decisions”, including but not limited to migration, public/private/dual practice preference, rural/urban preference, and clinical specialty. To meet “foundation year or internship experience” criteria for inclusion, papers had to focus on how doctors’ experience during foundation years or internship impacted their career intention and decisions. We defined internship as the period after primary medical qualification where doctors work in accredited positions in hospital settings to gain supervised experience, in line with another review (Zhao et al. 2021).

After de-duplication, two reviewers conducted two stages of independent screening (title and abstract, and full-text) and assessed selected papers for quality and relevance using two methods: (1) the Critical Appraisal Skills Programme (CASP) which includes 10 appraisal questions spanning recruitment strategy to ethical issues, and (2) the global categorization, described by Dixon-Woods et al (2007) (Malpass et al. 2009) which focuses on the “richness” of the findings and their contribution to the synthesis. The global categorization includes four categories: “key paper” (conceptually rich and could potentially make an important contribution to the synthesis), “satisfactory paper”, “irrelevant” to the synthesis, and methodologically “fatally flawed” (e.g. unclear study design and data source). “Irrelevant” and “fatally flawed” papers were excluded. We resolved disagreements on inclusion at all stages by discussion among the two reviewers.

### Translation and synthesis

We first used NVivo software (version 1.4) to facilitate close reading and extraction and comparison of concepts from each selected paper. Contextual information of all included papers was first extracted and all papers were read thoroughly by the same two reviewers. For further extraction we extracted first-order constructs (participants’ common-sense interpretation in their own words) and second-order constructs (researchers’ interpretation based on first-order constructs), and used both to develop third-order constructs (reviewers’ interpretation of original authors’ interpretation (Schutz 2012; Toye et al. 2014), as there were studies that did not primarily focus on linking internship experience and career outcomes but had relevant quotations and data supporting that link.

To identify initial constructs, four key papers were selected based on their richness and career outcomes examined. These were examined independently to identify and extract concepts from each paper, and discussed by two reviewers. The remaining papers were extracted by one reviewer and checked by another. Concepts from all included papers were then clustered into relevant categories through constant discussion and comparison. We then went back to the primary papers, and translated and compared our newly developed conceptual categories across all papers to ensure that all relevant data were represented. This translation process was conducted in Microsoft Word, with workings presented in the translation table (Appendix 3).

After completing the stages above, we organized the categories by higher-level themes to generate a visual conceptual model to illustrate how the internship experience influenced medical doctors’ career intention/decisions, i.e. our “line-of-argument”. We also applied the GRADE-CERQual framework, an approach to assess how much confidence to place in findings from qualitative evidence syntheses, to define our confidence at the category level (Lewin et al. 2018). The assessments were made by one reviewer and discussed with the other reviewer.

## Results

### Search results and article overview

**Figure 1:**
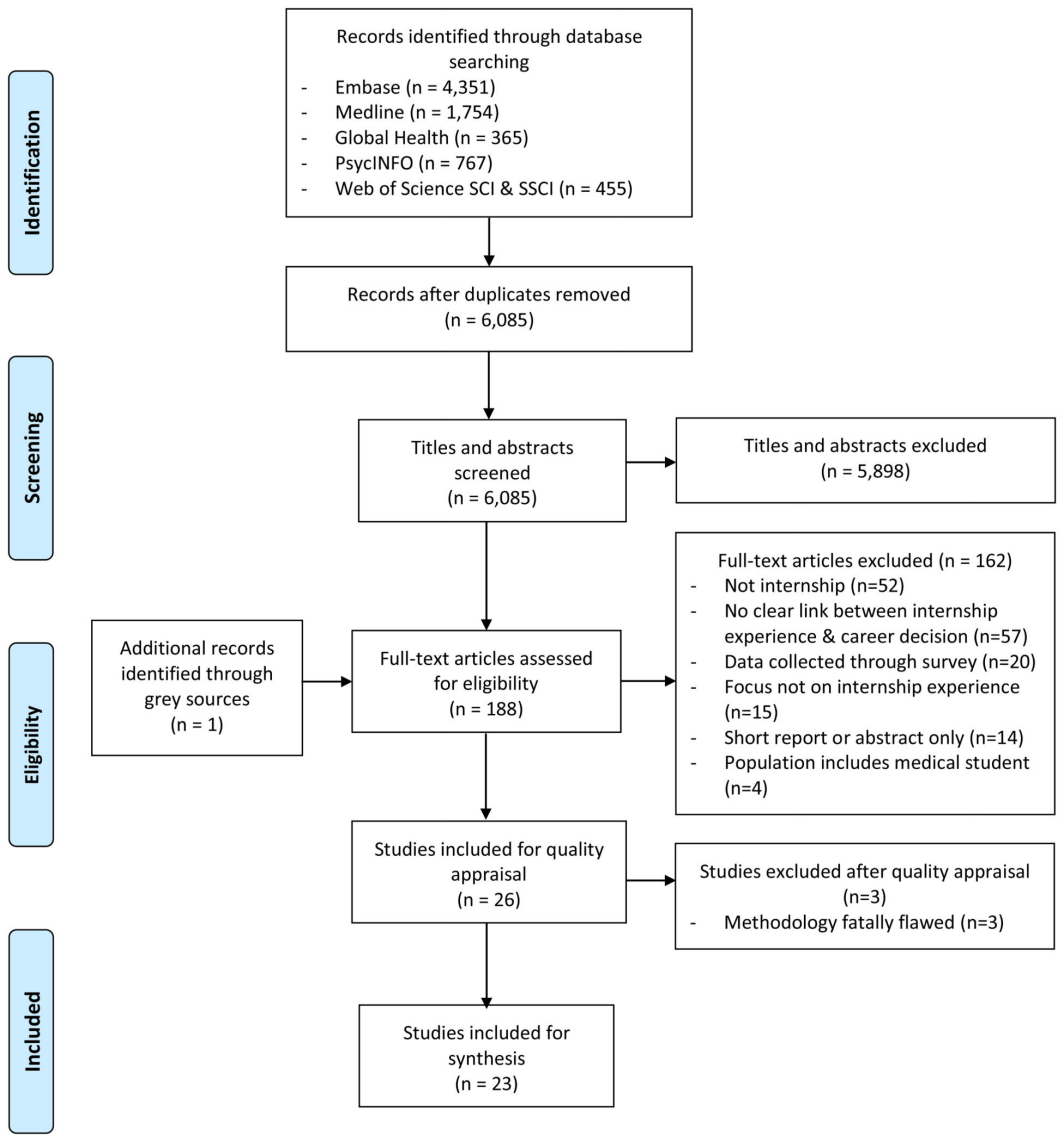
Preferred Reporting Items for Systematic Reviews and Meta-Analyses (PRIMSA) flowchart

Figure 1 summarizes the results of the search process. Of 6,085 citations identified after deduplication, 26 met inclusion criteria after the full-text review. Three further studies were excluded after quality appraisal, leaving 23 studies for the synthesis.

Table 1 provides an overview of included studies. Most studies included were from high-income countries, including 12 papers from the UK, two from Australia, and one each from New Zealand, Germany, Ireland and the Netherlands. Only three studies were conducted in low- and middle-income countries (LMICs); India (Sreedaran and Hegde 2018), Sierra Leone (Woodward et al. 2018) and South Africa (Essa 2011). Fourteen papers examined career intentions (current interns considering future choices) and nine examined career decisions (doctors retrospectively reflect how they made current career choices). In terms of the career outcome examined, 14 studies examined specialty choices (specifically six general practice, three psychiatry, one surgery and others without a specific specialty focus), three examined practice location (including two rural practice and one migration), three investigated general career intention, two investigated why doctors are not going straight into specialty training (i.e., taking an F3 year in the UK) and one examined leaving specialty training midway (i.e., women leaving surgery training in Australia and New Zealand (Liang et al. 2019)).

Most studies were assessed as good quality against the CASP checklist, however, most lacked a description of the relationship between researchers and participants (Q6). We also rated the papers based on their relevance as described by Dixon-Woods et al (Dixon-Woods et al. 2005): Twelve papers were rated as key papers and 11 as satisfactory.

We identified 14 categories of how internship experience influence doctors’ career intention/decision, further summarized into three high-level themes. Descriptions are provided below and in Table 2.

### Theme 1: Finding which career option is “more me”

The papers suggested that interns appraise the potential personal fit between their clinical interests and lifestyle, and to find the “more me” option among different specialties, different roles and positions, and different organizations.

#### The importance of hand-on experiences, real-life exposure (category 1) and positive experiences (category 2)

The papers drew attention to how the experience of employment gave interns real-life exposure of what it means to be a doctor (with significantly more clinical responsibility as compared with medical students, e.g. prescribing, communicating with patients and families). This appeared to enable them to realize what ‘fits’ with their personality (Scanlan et al. 2018; Woodward et al. 2018), what they enjoy doing (Williams and Cantillon 2000; Sreedaran and Hegde 2018; Woodward et al. 2018; Querido et al. 2018), and what is “more me” (Woodward et al. 2018), which influences career decision making (Essa 2011).

Importantly there were cases where interns felt poorly prepared for the tasks they encountered e.g. “not feeling confident managing very unwell people” and describing it as anxiety-inducing (Brodribb et al. 2016; Spooner et al. 2017), or not feeling adequately prepared to enter specialty training (Smith et al. 2018). Some studies also suggested that interns realized their responsibilities were quite different from experiences as a student forcing some to reconsider their career choices (Appleton et al. 2017; Harris et al. 2020).

*“I hated my job [psychiatry] but I loved it as a student… I just found it really depressing as a doctor whereas as a student I found all the stories really interesting.”*

*[GP0P18-(Spooner et al. 2017)]*

Positive experiences in managing patients, making decisions regarding their care, and seeing positive results helped boost interns’ confidence and sense of readiness (Scanlan et al. 2018; Ludwig et al. 2018) and inspired some to develop skills in areas they felt inadequately prepared for by medical school (Appleton et al. 2017). In some cases, interns could also rebuild their previously damaged confidence with a supportive team and positive experiences (Parker et al. 2014; Spooner et al. 2017), as an example of positive experience:

*“It’s a decision that I don’t think I would’ve made. Actually thinking back to making that decision, knowing how confident I was that I enjoyed anaesthetics; it still felt like a big thing to click that button and apply for that job. I most definitely wouldn’t have done that if I hadn’t had that experience in anaesthetics.”*

*[Story 2 Steven-(Scanlan et al. 2018)]*

#### Considering work-life balance (category 3) and wellbeing (category 4)

Internship exposes individuals to the workloads and working hours of different rotations. The ability to control working hours was a considerable influence on their career decision. Most studies reported having a manageable work-life balance and specialties that fit with lifestyle aspirations as having considerable influence on career decisions, which also had a gender aspect to it, e.g., having children and raising a family (Williams and Cantillon 2000; Merrett et al. 2017; Sreedaran and Hegde 2018; Scanlan et al. 2018; Woodward et al. 2018; Querido et al. 2018). Interns seeking a good work-life balance and the flexibility to pursue interests outside of work seemed to be drawn to specialties that had more regular working hours (Beattie et al. 2017; Sreedaran and Hegde 2018; Ludwig et al. 2018) and some interns stayed away from specialties they loved and had a passion for because the demands of work conflicted with their personal desire to achieve a proper work-life balance (Spooner et al. 2017). Internship experience in rural areas was attractive to some interns because of the rural lifestyle, perceived lower stress and greater ability to maintain work-life balance (Cuesta-Briand et al. 2020). As an example for work life balance:

*“I would love to do gastroenterology, but… I just know I wouldn’t have a good work-life balance. Work-life balance is really important to me, I’d probably say more so than what I want to do in my career… if I’m not enjoying myself out of work, it’s just not worth it for me.”*

*[GP0P10-(Spooner et al. 2017)]*

Linked to the working hours and work-life balance were exhausting and stressful rotations. Some were described as being a treadmill with no breaks contributing to burnout and impacting wellbeing (Scanlan et al. 2018). Others (e.g., general practice) were described as being lonely with no support and team spirit (Merrett et al. 2017). Psychiatry was seen as emotionally draining, with some interns casting doubts on whether they saw themselves working in that field in the future (Beattie et al. 2017).

*“When you’re with a patient and in particular a depressed patient…you feel emotionally drained at the end of it so I don’t know if I could cope with doing that all day for the rest of my life.”*

*[Participant 10-(Beattie et al. 2017)]*

#### The role of self-identity (category 5)

Although only reported in a few papers, interns also reflected on whether their proposed career, as well as its status and public image, fitted with their self-identity and whether they would be considered a ‘proper doctor’ by choosing these specialties. For instance, they were concerned about being deskilled by choosing to pursue psychiatry because of not dealing with physical health problems (Appleton et al. 2017). General practice was perceived to attract less respect and a negative public image because of lack of awareness of what GPs do (Spooner et al. 2017; Alberti et al. 2017). On the other hand, specialties such as surgery were considered to be of a higher status and power (Olsson et al. 2019).

*“I don’t care what my friends and family think, it’s the wider population. … I had the girl doing my nails one day. She said, ‘Oh, what kind of doctor are you going to be?’ I said, ‘I’m going to be a GP’. And she said, ‘Oh, do you have to go to medical school for that?’ And I just thought, … there’s just that a bit less respect, isn’t there, than, ‘Oh yes, I’m a brain surgeon.”*

*[GP1P16-(Spooner et al. 2017)]*

### Theme 2: Exploring, experiencing, and witnessing workplace and organizational norms

Through working with people including their direct supervisors/consultants, peers, senior colleagues including medical and nursing staff as well as with the local community, interns got to explore, experience, and witness workplace and organizational norms of medical facilities. This also helped them decide on which specialty, organization and work locations are preferable.

#### Relationship with consultants (category 6), peers (category 7), senior colleagues and the team (category 8)

The findings demonstrated that consultants and supervisors had a considerable influence on career choice (Croghan and Baker 2020). Supervisors as role models moulded interns’ attitudes, making them feel valued and supported (Spooner et al. 2017; Scanlan et al. 2018) and inspiring interns to want to be like them (Parker et al. 2014; Alberti et al. 2017; Querido et al. 2018). Some consultants also lobbied interns towards their specialty (Olsson et al. 2019). In contrast, bullying from supervisors or lack of role models to look up to were reported to contribute to interns shying away from or pausing a career in those specialty areas or work locations (Smith et al. 2018; Liang et al. 2019).

Building relationships with peers and other team members also played a vital role in enriching interns’ experiences. Positive workplace relationships with other seniors including medical staff and nursing staff influenced career decisions, as they acted as a source of information on training and work opportunities (Cuesta-Briand et al. 2020). Interns also sought information from colleagues and from social events organised by specialty unions, career workshops and shadowing doctors to inform their career decision (Spooner et al. 2017; Sreedaran and Hegde 2018; Querido et al. 2018; Olsson et al. 2019; Cuesta-Briand et al. 2020). In some other cases, interns witnessed their specialty registrars being “broken”, or felt unwelcomed or unsupported by the team, or were blamed by seniors when things went wrong, and thus switched their career plans to a different specialty (Spooner et al. 2017; Scanlan et al. 2018). As an example of relationship challenges with nursing staff:

*“The nursing staff wouldn’t listen to me. They would then go and get consultants and run everything I did past them. There are some strong characters in that department and it’s well known that that is the case. I just clashed with them and I find it quite condescending and made me feel like, that you weren’t a doctor, that you are more a medical student because everything you said had to be verified by a consultant.”*

*[P9-(Smith et al. 2018)]*

Competition between peers put some interns off certain specialties, especially when it was felt to be unhealthy and constraining (Olsson et al. 2019).

*“‘At the surgical and orthopaedic departments where I’ve been, you need to be fairly assertive, even bullish, to somehow get the educational experience you need. You have to make sure you get into theatre, struggle, really, and hinder others in your way to becoming a specialist. And that was not something I had any desire to do. Being somewhere where there was a lot of competition, I wasn’t interested in that. At all’.*

*[No. 5, internal medicine, woman-(Olsson et al. 2019)]*

#### Relationship with the community (category 9)

Another key category emerged on relationship with the community. Through working with patients and the community, interns were able to develop close doctor-patient relationships and felt integrated into the community (Querido et al. 2018). These experiences made interns appreciate the psychosocial aspects of patient care (Appleton et al. 2017) and gave them a sense of helping the community and changed perceptions of where they would want to work (Brodribb et al. 2016; Ludwig et al. 2018).

#### Characteristics and hierarchy of career options and specialties (category 10)

Often in their interaction with consultants and other senior colleagues, interns would hear them commenting on other specialties, sometimes stating that some are inferior (Firth and Wass 2011; Merrett et al. 2017; Harris et al. 2020; Croghan and Baker 2020). The interns themselves also developed attitudes towards certain specialties interacting with perception of self-identity as discussed previously. For instance, when discussing their career interests with their senior clinicians, “just a GP” or “too good for GP” were often used to create the impression that general practice was inferior, unworthy, unexciting or a reserve option (Spooner et al. 2017; Alberti et al. 2017). Interns seeking to specialize in psychiatry also described being stigmatized.

*“There is always a stigma. In my medicine F2 post … I had just finished psychiatry and when I said that I wanted to do psychiatry my consultant said something like ‘then there is no point bothering with you then’”*

(Appleton et al. 2017)

Nonetheless, some interns perceived such comments as “banter”, and a natural consequence of consultants’ passion for their specialty.

*“ ‘I think there are certain specialties that get made fun of. It hasn’t really had any impact on my decision making. I think probably because they get made fun of, partly, because people are jealous.”*

*[FD24, anaesthetics-(Harris et al. 2020)]*

#### Workplace resources, environment (category 11) and feeling valued by the organization and healthcare system (category 12)

Interns were drawn to workplace environments with sufficient resources, that were supportive and seen to have high morale. Poor rest facilities, limited access to catering facilities and limited parking contributed to feelings of being unappreciated. This was compounded by the pressures from workloads, to cover rota gaps and from working in poorly resourced facilities (Rizan et al. 2019; Hollis et al. 2020). In some cases interns felt undervalued and disengaged leading to frustrations with the whole system (Scanlan et al. 2018). They described being viewed as “ward mules” used for “service provision” (Rizan et al. 2019) or the “cheapest option” to fill rota holes and therefore felt only valued for the heavy workload they carry (Hollis et al. 2020).

*“…So the thought of having to do six, seven years to consultant, there’s no way. There’s literally no way I would do it. Management don’t know who I am, don’t know what I’m about. And if I raise a concern, I think they see that you’re a hassle, it’s a problem, as opposed to, you’re a valued team member that they think is worth being there. I don’t, yes, I don’t feel valued in that capacity.*

*[Story 5 Clare-(Scanlan et al. 2018)]*

Additionally in one study from South Africa, interns described frustrations with the public health sector, especially regarding management’s misappropriation of funds meant to provide essential resources and equipment in hospitals leading to frequent stockouts, thus *“that’s why everybody is leaving”* (Essa 2011). This could be a specific issue for LMICs.

### Theme 3: Worry about the future

Aside from personal best fit and the norms of the current workplaces, interns also considered the future implication of their possible career choices, especially whether they would get a job and whether they would advance their career from a training and professional development perspective.

#### Job market policies and changes (category 13)

Job market policies like wages and employment contracts, and interns’ perceptions about the future job market influenced their career choices. Interns described choosing specialties strategically based on existing workforce gaps or a growing need (e.g. “more employable” (Woodward et al. 2018)), or on the contrary after witnessing senior doctors encounter a lack of job opportunities and being disillusioned (Croghan and Baker 2020). In some contexts (e.g. UK) changes in junior doctors’ contracts also made alternative specialist training unattractive compared to General Practice because the latter has a shorter training period. Changes in employment contracts for junior doctors also made interns consider migrating to work in other countries where they would strike a good work-life balance with better working conditions (Smith et al. 2018). However, the influence of job market policies and changes may be limited as they are sometimes difficult to predict (Croghan and Baker 2020).

#### Future training and professional development opportunities (category 14)

Interns described an interest to pursue specializations where there was more 1:1 mentorship and training, as well as commitment from the seniors and the organization towards professional development, (Smith et al. 2018; Scanlan et al. 2018) instead of specializations where they perform mundane administrative tasks (Hollis et al. 2020). On some other occasions, interns with an interest to work in rural areas were frustrated by lack of training pathways and future development opportunities if they do not want to proceed with a GP pathway (Cuesta-Briand et al. 2020):

*“I actually find it very disappointing after working in rural areas and wanting to go back to those areas so badly, that unless you specifically want to be that rural GP, there’s firstly no pathway. And two, it’s not only not encouraged, it’s almost frowned upon. I find it amazing because the whole time I was in rural areas people talk about how much they’re trying to bring people rurally. When I look at it I kind of see a lot of closed doors.”*

*[I19; Male; FGY1-(Cuesta-Briand et al. 2020)]*

### Confidence in findings

Details of our GRADE-CERQual assessment are provided in Table 2 and Appendix 3. The assessment of confidence applies only to the 14 categories. We rated 8 categories of high confidence and 5 moderate confidence. The “image of professions and self-identify” was rated very low confidence due to concerns about data adequacy and relevance.

## Discussion

**Figure 2:**
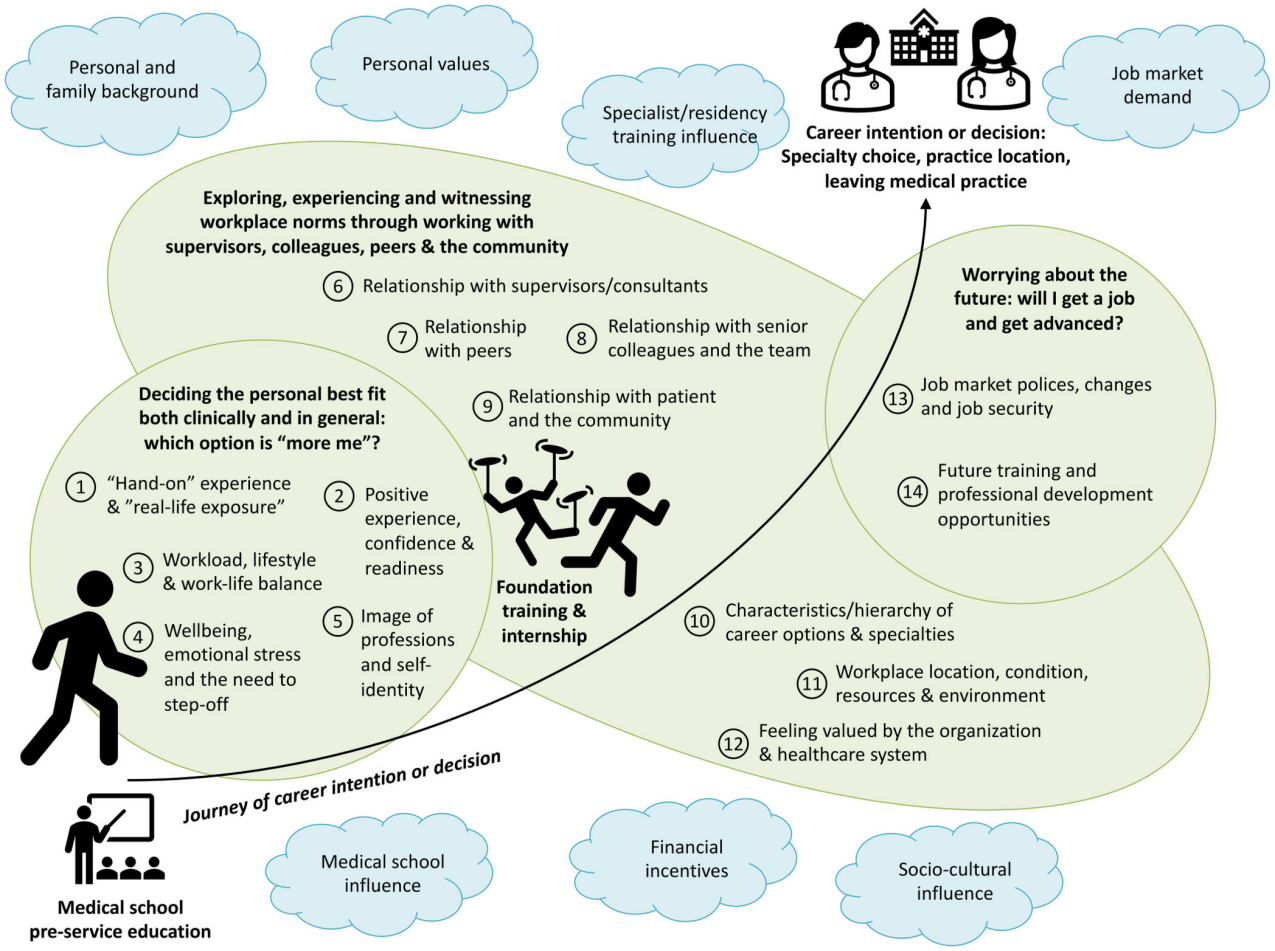
Line of argument Note: The final phase of meta-ethnographic analysis is to develop a conceptual model or line of argument that is abstracted from, but more than the sum of, the themes. This figure illustrates our conceptual model which is the journey of career intention decision-making and how foundation years and internship influence such journeys. These junior doctors’ intentions may be shaped early during medical school training, this can be cemented or changed during the chaotic and stressful foundation year and internship training period, which is the focus of our synthesis. Foundation doctors and interns need to decide on the “more me” option of possible career choices (category 1-5), draw on experience in the workplace and relationships with different groups of people (category 6-12), and also think about the future implications of career options (category 13-14). These categories are intertwined and overlapping. We also acknowledged many other factors including personal and family background and personal values may play a significant role in the career-decision making, and doctors could further change their mind during specialist training; moreover, the career decision is also influenced by the job market and employment terms and conditions. These factors extracted from previous reviews are represented (in “blue clouds”) outside of the main categories identified by this review, though it should be noted that this is by no means an exclusive list of factors that influence career intention or decision.

Our meta-ethnographic review summarized how experiences during internship appear to significantly influence medical doctors’ career intentions and decisions. We developed a conceptual model to illustrate the journey of career intention/decision making during this period (Figure 2). Our synthesis highlights that improving internship experiences is a broad agenda. It especially spans ensuring sufficient and positive clinical exposure while maintaining work-life balance and wellbeing, improving workplace environments and culture, building a supportive relationship with consultants and other medical and nursing team members, and relieving worries about future job security and professional development following the internship period.

While our review focused only on qualitative studies, the impact of internship experience on career decisions is also evidenced by quantitative studies. A survey in Ireland suggested that negative experience as an intern, especially burnout and callousness, was significantly associated with doctors’ intention to leave the country permanently (Cronin et al. 2019). Another study in Australia that surveyed medical, nursing, pharmacy and allied health students also confirmed that students satisfied with their rural placement (16-20 weeks for medicine and allied health students and 2-8 weeks for nursing and pharmacy) were twice as likely to consider living and working in a regional, rural or remote location following graduation, after adjusting for other demographic covariates (Fatima et al. 2018).

Our findings also align with the wider literature on career decisions and identity work. The social cognitive career theory by Lent et al. (1994) suggests the influence of personal, environmental and learning experiences and the interaction between these as well as contexts that shaped personal agency, formulation, pursuit and attainment of career goals and also performance (Lent et al. 1994; Lent and Brown 1996). Identity theories especially “possible selves” (Markus and Nurius 1986) and “provisional selves” (Ibarra 1999) further illustrate how the decisions are made between different career options. Interns observe role models (consultants, or other senior colleagues) to identify potential identities. They then experiment and try on different career options throughout different rotations to inform their career intentions and who they want or fear of becoming, i.e. “possible selves” (Markus and Nurius 1986). The theory of “provisional selves” further describes how these possible professional identities are formed, and the process may also include self-evaluation (internal self-congruence) and perceptions of external judgment from either peers, colleagues or society (Ibarra 1999). This is in line with our findings especially as interns figuring out their self-identity, relationship with other colleagues and seniors as well as absorbing comments from others on the characteristics and hierarchy of career options and specialties.

Our study has important implications for policy and practice. While internship trainings are context dependent, the following practices should be considered and tested to understand their impact on career planning. Medical regulators and training institutions should ensure interns have sufficient exposure to different career options including specialty rotations and work locations to account for varied preferences. The internship environment should also be regularly monitored to ensure adequate resources are available (Zhao et al. 2022). This exposure should allow interns to explore different potential career interests and whether they match their clinical and lifestyle expectations, e.g. incorporating rural clinical rotations may attract junior doctors into rural and remote area practice (World Health Organization 2021); arranging short specialty tasters or shadowing senior colleagues may interest junior doctors in specialties with recruitment difficulties (East Anglian Foundation Programme 2010). Changes in contractual, job market and future training policies should also be clearly communicated well in advance, by policy makers, regulators and training institutions, to clearly signal upcoming opportunities and alleviate stress about future job security.

Most studies did not report interns receiving formal advice from career advisors, but consultants and other senior colleagues seem key sources of information (Spooner et al. 2017; Sreedaran and Hegde 2018; Smith et al. 2018; Cuesta-Briand et al. 2020). Resources, guidance and training should be provided to senior healthcare professionals so they can ensure good mentoring practices, like having regular one-to-ones with trainees focusing not only on educational and academic progress but also on professional and personal growth (Stamm and Buddeberg-Fischer 2011; Han et al. 2014), refraining from undermining specific specialities and incorporating this into faculty development (NHS Health Education England and Medical Schools Council 2016; Alberti et al. 2017) and learning how to best support junior doctors’ career decision-making processes. Lastly, sufficient resources like rest and catering facilities and a positive and supportive working environment should also be provided so that interns feel valued, instead of just being made to feel like “cheap labour”. This could be as simple as asking consultants and colleagues to remember the names of their trainees (Cleland et al. 2018; Scanlan et al. 2018).

Several limitations should be considered for this review. To start with, as meta-ethnography is an interpretative approach to qualitative evidence synthesis, a different research team might provide a different interpretation of the data. In our case we re-interpreted some first-order constructs when the paper did not primarily focus on linking experience and career outcomes but where we found relevant data. While we did not contact the original teams and clarify such re-interpretations, the extraction and interpretation of concepts were led by one author and reviewed by another, and further shared and discussed within the whole research team for validation and feedback. Second, we had a very strict definition of internship experience during the screening process and excluded papers focusing on residency unless the papers explicitly stated that the focus was on the first year of residency which is commonly referred to as internship. This resulted in our not including any paper published from the US and other countries where the internship year is embedded in multi-year specialty residency programs, thus our findings might be less applicable to those settings. Third, we acknowledge that both internship and career preference are context-dependent and vary by the socio-cultural environment of the geographical locations in which they are situated and the health system structure. For example in some countries including the UK, the choice to enter the private sector is much more constrained especially for early career doctors as post-graduate training opportunities are limited to public sector, and also “rural” practice in high-income countries could be quite different from many LMIC settings which may be more under-resourced and lacking basic infrastructure (Lehmann et al. 2008); Additionally much of our literature is from a small set of countries - for example, only three of our included papers are from LMICs which reflects the paucity of data from regions where the internship experience could be most stressful and from which many junior doctors choose to migrate after internship (Essa 2011; George and Reardon 2013). Future work is needed to investigate internship experience and career outcomes in different settings and additional factors linking internship experience and career intention/decision. Lastly, while our study focused on a broad list of career outcomes, we did not explicitly differentiate career intentions and career outcomes. Interns could choose a different specialty than their intention due to job market demand or training availability, and such decision-making is dynamic and could change even during specialty training. Our conceptual model (Figure 2) recognized such limitations and highlighted job market demand and other factors that influence career choices identified in previous systematic reviews. We recommend future studies explore whether our conceptual model is appropriate across different countries and contexts, including for different modes of internship (e.g. in the US) while extending research to other factors that might influence career outcomes such as personal values, medical school experiences and job market demands, and other career outcomes for example non-patient facing specialties, research and management.

## Conclusion

Our meta-ethnographic review revealed a range of ways in which internship experience shapes medical doctors’ career intentions/decisions. Medical regulators and educators, hospital administrators, and policymakers need to take these into consideration to improve the training experience for these junior doctors generally, but also use to inform efforts to attract doctors to less popular specialties or work locations where they are most needed. We propose our conceptual model should be further tested in new research across different contexts.

## Supplementary Material

Appendix 1

Appendix 2

Appendix 3

Appendix 4

## Figures and Tables

**Table 1:** Characteristics of included studies

Author & year	Country of study	Study population	Study sample size	Study aim	Data collection methodology	Career outcome	Career intention or decision	CASP score (out of 20)	Assessment
(Alberti et al. 2017)	UK	General Practice Specialty Registrars	49	Understand comments made in clinical settings to GP trainees about GP	Mixed methods - questionnaire survey, focus group discussions	Specialty choice - general practice	Decision	16	satisfactory
(Appleton et al. 2017)	UK	Psychiatry trainee	21	Explore factors that influence attitudes towards mental health and a career in psychiatry	Qualitative - in-depth interviews	Specialty choice - psychiatry	Decision	20	key
(Beattie et al. 2017)	UK	Foundation year 1 doctors	14	Understand lived experience of psychiatry placement and how job components influence attitudes	Qualitative - semi-structured interviews	Specialty choice - psychiatry	Intention	20	satisfactory
(Brodribb et al. 2016)	Australia	Junior doctors graduated between 1-6 years	41	Understand the effect of rural placements on perception on working in rural area	Qualitative - semi-structured interviews	Practice location - rural practice	Decision	18	satisfactory
(Croghan and Baker 2020)	Ireland	Interns	5	Understand factors influencing specialty choices of medical students	Mixed methods - questionnaire survey, focus group discussions	Speciality choice	Intention	16	satisfactory
(Cuesta-Briand et al. 2020)	Australia	Postgraduate year 1-5 junior doctors	21	Understand factors influencing career decision-making with respect to specialty area and rural location	Qualitative - semi-structured interviews	Practice location - rural practice	Decision	17	key
(Essa 2011)	South Africa	Doctors who completed internship up to 8 months	10	Gain insight into the medical internship process in South African state training hospitals	Qualitative - semi-structured interviews	General career choice	Intention	17	key
(Firth and Wass 2011)	UK	Foundation year 2 doctors	25	Understand perceptions of general practice attachment	Qualitative - semi-structured interviews and focus group discussions	Specialty choice - general practice	Intention	16	satisfactory
(Harris et al. 2020)	UK	Foundation year 2 doctors	24	Investigate what influences decision-making about general practice as possible career	Qualitative - semi-structured interviews	Specialty choice - general practice	Intention	19	key
(Liang et al. 2019)	Australia and New Zealand	Women who chose to leave surgical training	12	Understand reasons for women to leave surgical training with a feminist lens	Qualitative - interviews	Others - leaving specialty training	Decision	19	satisfactory
(Ludwig et al. 2018)	Germany	Practice year students	19	Understand barriers of practice year students with GP tertiary electives	Qualitative - guided interviews	Specialty choice - general practice	Intention	16	satisfactory
(Merrett et al. 2017)	UK	Foundation year 1 doctors	74	Understand attitude of newly qualified doctors towards GP career	Qualitative - focus group discussions	Specialty choice - general practice	Intention	16	satisfactory
(Olsson et al. 2019)	Sweden	Young doctors undertaking specialty training	15	Understand the processes preceding specialty choices and the influence of perceived status and other symbolic capital on their choices	Qualitative - in depth semi-structured interviews	Speciality choice	Decision	17	key
(Parker et al. 2014)	New Zealand	Final year undergraduate medical students	11	Explore factors that influence attitudes towards general practice as a career	Qualitative - focus group discussions	Specialty choice - general practice	Intention	20	key
(Querido et al. 2018)	Netherlands	Final transition year students	24	Understand factors influencing career choices of medical students in final study year	Qualitative - in depth semi-structured interviews	General career choice	Intention	20	satisfactory
(Rizan et al. 2019)	UK	Foundation year 3 doctors	14	Explore the reasons why doctors are choosing to take a year out of training and impact upon future career choices	Qualitative - in depth semi-structured interviews	Others - not go straight into specialty training	Decision	18	key
(Scanlan et al. 2018)	UK	Foundation year 2 doctors	21	Understand Foundation year 2 doctors’ workplace support and its influence on career intentions	Qualitative - semi-structured interviews	General career choice	Intention	20	key
(Smith et al. 2018)	UK	Foundation year 1 doctors	17	Explore reasons for doctors to leave UK medicine	Qualitative - semi-structured interviews	Practice location - migration	Intention	20	key
(Spooner et al. 2017)	UK	Foundation year 2 doctors	20	Understand workplaces, practices and colleagues’ effect on doctors’ career decision	Qualitative - narrative interviews	Speciality choice	Intention	18	key
(Sreedaran and Hegde 2018)	India	Psychiatry residents	17	Explore reasons for choosing psychiatry for future specialization	Qualitative - focus group discussions and in-depth interviews	Specialty choice - psychiatry	Decision	16	satisfactory
(Hollis et al. 2020)	UK	Foundation year 2 doctors	16	Explore the reasons for foundation doctors choosing not to go straight into specialty training	Qualitative - semi-structured interviews	Others - not go straight into specialty training	Decision	20	key
(Williams and Cantillon 2000)	UK	Pre-registration house officers	15	Explore female pre-registration house officers views of surgery as a possible career	Qualitative - in depth semi-structured interviews	Specialty choice - surgery	Intention	17	key
(Woodward et al. 2018)	Sierra Leone	Junior doctors	15	Explore the career aspirations and specialist training preferences and choices	Qualitative-in-depth interviews, digital diaries	Speciality choice	Intention	14	satisfactory

**Table 2:** CERQual Summary of Qualitative Findings

Categories	Studies contributing to the review findings	Overall CERQual assessment	Explanation of judgement
**Theme 1: Finding which career option is “more me”**			
**1. The “hand-on” experience and “real life” exposure**	16/23 (Williams and Cantillon 2000; Firth and Wass 2011; Essa 2011; Merrett et al. 2017; Spooner et al. 2017; Alberti et al. 2017; Appleton et al. 2017; Sreedaran and Hegde 2018; Smith et al. 2018; Scanlan et al. 2018; Ludwig et al. 2018; Woodward et al. 2018; Querido et al. 2018; Rizan et al. 2019; Harris et al. 2020; Croghan and Baker 2020)	High confidence	Only minor methodological concerns on relationship between the researcher and participants in a number of studies, and in another few studies the recruitment and analysis processes were not clearly described
**2. Positive experience, confidence and readiness**	9/23 (Parker et al. 2014; Brodribb et al. 2016; Spooner et al. 2017; Appleton et al. 2017; Smith et al. 2018; Scanlan et al. 2018; Ludwig et al. 2018; Harris et al. 2020; Hollis et al. 2020)	Moderate confidence	Very minor methodological concerns on relationship between the researcher and participants in a few studies, moderate concerns regarding relevance as all reported from HIC and no LMIC data
**3. The workload, worklife balance, lifestyle**	16/23 (Williams and Cantillon 2000; Essa 2011; Merrett et al. 2017; Beattie et al. 2017; Spooner et al. 2017; Appleton et al. 2017; Sreedaran and Hegde 2018; Smith et al. 2018; Scanlan et al. 2018; Ludwig et al. 2018; Woodward et al. 2018; Querido et al. 2018; Rizan et al. 2019; Cuesta-Briand et al. 2020; Croghan and Baker 2020; Hollis et al. 2020)	High confidence	Only minor methodological concerns on relationship between the researcher and participants in a number of studies, and in another few studies the analysis processes were not clearly described; and minor concerns regarding coherence
**4. Well-being, emotional stress and the need to step off**	9/23 (Essa 2011; Merrett et al. 2017; Beattie et al. 2017; Appleton et al. 2017; Smith et al. 2018; Scanlan et al. 2018; Rizan et al. 2019; Harris et al. 2020; Hollis et al. 2020)	Moderate confidence	Moderate concerns on relevance as all reported from HIC, the one paper from South Africa did not explicitly relate this with career decision/intention
**5. Image of professions and self-identity**	7/23 (Williams and Cantillon 2000; Merrett et al. 2017; Beattie et al. 2017; Spooner et al. 2017; Alberti et al. 2017; Appleton et al. 2017; Olsson et al. 2019)	Very low confidence	Serious concerns on adequacy as studies only offered thin or very thin data; Serious concerns on relevance as all studies reported specialty choices only, not clear if it is directly relevant to internship experience and that all studies from HIC, no LMIC data
**Theme 2: Exploring, experiencing, and witnessing workplace and organizational norms**			
**6. Relationship with supervisors/consultant s**	16/23 (Essa 2011; Parker et al. 2014; Brodribb et al. 2016; Merrett et al. 2017; Spooner et al. 2017; Alberti et al. 2017; Appleton et al. 2017; Sreedaran and Hegde 2018; Smith et al. 2018; Scanlan et al. 2018; Woodward et al. 2018; Querido et al. 2018; Liang et al. 2019; Rizan et al. 2019; Olsson et al. 2019; Croghan and Baker 2020)	High confidence	Only minor methodological concerns on relationship between the researcher and participants in a number of studies, and in another few studies the recruitment and analysis processes were not clearly described
**7. Relationship with peers**	7/23 (Smith et al. 2018; Woodward et al. 2018; Querido et al. 2018; Rizan et al. 2019; Olsson et al. 2019; Cuesta-Briand et al. 2020; Hollis et al. 2020)	High confidence	Only minor methodological concerns on relationship between the researcher and participants in a number of studies, and in another few studies some other aspects were not clearly described; minor concerns regarding relevance as most but one paper are from HIC
**8. Relationship with senior colleagues and the team**	12/23 (Parker et al. 2014; Merrett et al. 2017; Spooner et al. 2017; Alberti et al. 2017; Sreedaran and Hegde 2018; Smith et al. 2018; Scanlan et al. 2018; Querido et al. 2018; Rizan et al. 2019; Cuesta-Briand et al. 2020; Harris et al. 2020; Croghan and Baker 2020)	High confidence	Only minor methodological concerns on relationship between the researcher and participants in a number of studies, and in another few studies the recruitment, ethics and analysis processes were not clearly described
**9. Relationship with patient and the community**	6/23 (Brodribb et al. 2016; Merrett et al. 2017; Appleton et al. 2017; Ludwig et al. 2018; Woodward et al. 2018; Querido et al. 2018)	Moderate concerns	Minor methodological concerns as in a few studies the design, recruitment, ethics and analysis processes were not clearly described; minor concerns on adequacy as studies offered moderately rich to very thin data; minor concerns regarding relevance as most but one paper are from HIC
**10. Characteristics and hierarchy of career options and specialties**	10/23 (Williams and Cantillon 2000; Firth and Wass 2011; Merrett et al. 2017; Spooner et al. 2017; Alberti et al. 2017; Appleton et al. 2017; Woodward et al. 2018; Liang et al. 2019; Harris et al. 2020; Croghan and Baker 2020)	Moderate confidence	Minor methodological concerns as in a few studies the design, recruitment, ethics and analysis processes were not clearly described; minor concerns on coherence; Moderate concerns regarding relevance as all studies reported specialty choices only, not clear if this will be relevant to other career options and most reported from HIC, one LMIC data
**11. Workplace location, condition, resources and environment**	6/23 (Essa 2011; Sreedaran and Hegde 2018; Smith et al. 2018; Rizan et al. 2019; Cuesta-Briand et al. 2020; Hollis et al. 2020)	Moderate concerns	Minor methodological concerns as in a few studies the design, recruitment, ethics and analysis processes were not clearly described; minor concerns on adequacy as studies offered moderately rich to very thin data
**12. Feeling valued by the organization and healthcare system**	6/23 (Essa 2011; Smith et al. 2018; Scanlan et al. 2018; Rizan et al. 2019; Cuesta-Briand et al. 2020; Hollis et al. 2020)	High confidence	Minor methodological concerns as in a few studies the design, recruitment, ethics and analysis processes were not clearly described; minor concerns regarding relevance as most but one paper are from HIC
**Theme 3: Worry about the future**			
**13. Job market polices and changes, job security: will I get a job**	9/23 (Merrett et al. 2017; Spooner et al. 2017; Alberti et al. 2017; Smith et al. 2018; Woodward et al. 2018; Rizan et al. 2019; Cuesta-Briand et al. 2020; Croghan and Baker 2020; Hollis et al. 2020)	High confidence	Only minor methodological concerns as in another few studies the recruitment, ethics and analysis processes were not clearly described; and minor concerns on coherence
**14. Future training and professional development opportunities: will I get advanced**	8/23 (Essa 2011; Parker et al. 2014; Merrett et al. 2017; Smith et al. 2018; Scanlan et al. 2018; Querido et al. 2018; Cuesta-Briand et al. 2020; Hollis et al. 2020)	High confidence	Only minor methodological concerns as in another few studies the recruitment, ethics and analysis processes were not clearly described

## Practice points

- Medical regulators and educators, hospital administers, and policymakers should ensure positive and inspiring clinical exposure for interns and also sufficient exposure to different context-relevant career options including specialty rotations and work locations to account for varied preferences.
- Sufficient resources like rest and catering facilities and a positive and supportive working environment should also be provided so that interns feel valued.
- Changes in contractual, job market and training policies should be clearly communicated early to relieve doctors’ worries about their future.
- Resources, guidance and training should be provided to senior healthcare professionals so they can ensure good mentoring practices, refrain from undermining specific specialities and learn how to best support junior doctors’ career decision-making processes.
- Future studies should explore whether our conceptual model is appropriate across different countries and contexts, including for different modes of internship (e.g. in the US) and different career outcomes.
