# Supplementary material for: How do foundation year and internship experience shape doctors’ career intentions and decisions? A meta-ethnography
Source: Med Teach. Author manuscript; Available in PMC 2024 Jan 19. (PMC7615548; doi:10.1080/0142159X.2022.2106839)
Supplement: Appendix 1 [file EMS193419-supplement-Appendix_1.docx]

**Appendix 1. Synthesis method, search strategy and papers excluded at full-text stage**

### Getting started (stage 1)

There are different methods for qualitative evidence synthesis. We chose meta-ethnography as we hoped to develop conceptual understanding through a process of constant comparison of concepts and metaphors, rather than to simply aggregate findings (17). At the end of the process, we aimed to develop a “line of argument” to make “a whole into something more than the parts alone” using a storyline and conceptual model (17,18).

### Deciding what is relevant (stage 2)

We conducted a systematic search using MEDLINE, Embase, Global Health, PsycINFO, Science Citation Index Expanded and Social Sciences Citation Index to identify relevant articles. We combined terms and phrases related to foundation doctors, interns, junior doctors, career choices (general terms, specialty choices, migration, rural, public or private sector etc.) and qualitative studies filters (19). We included papers published between 2000 and 2020 in English. The search strategy is provided in appendix 1.

We adopted a broad definition of career intention and decisions including but not limited to migration, public/private/dual practice preference, rural/urban preference, and general practice or specific specialty. To meet “foundation year or internship experience” criteria for inclusion, papers had to focus on how the experience during foundation years or internship impacted their career intention and decisions. We defined internship as the period after primary academic qualification where doctors work in accredited positions in hospital settings to gain supervised experience, in line with another review (1). We excluded papers relating to undergraduate medical education, clerkship and residency unless the papers explicitly stated that the focus is on the first year of residency which is commonly referred to as internship.

After de-duplication, we imported the citations into Abstrackr for the initial title and abstract screening (20). YZ reviewed all the titles and abstracts to assess eligibility for full-text review, and a random subset of 30% was reviewed by DM. The agreement rate on which study to include at this stage was high (percentage agreement 0.97 [excellent], Cohen’s Kappa 0.56 [moderate], Gwet’s AC1 0.97 [excellent]) thus we proceeded with the full-text review. All full texts were reviewed by YZ and DM independently and discussed for inclusion or exclusion. Additionally, all the included papers after the full-text stage were assessed using two methods of appraisal by YZ and DM individually: (1) the Critical Appraisal Skills Programme (CASP) (21), and (2) the global categorization, described by Dixon-Woods et al (18): “key paper” (conceptually rich and could potentially make an important contribution to the synthesis), “satisfactory paper”, “irrelevant” to the synthesis, and a methodologically “fatally flawed” (for example unclear study design and data source, or using a quantitative numeric and counting analytical approach to qualitative interviews data and unable for further synthesis). We excluded all “irrelevant” papers at full-text stage and “fatally flawed” papers at the quality appraisal stages, but did not exclude papers that were assessed low quality based on the CASP checklist unless they were assessed as fatally flawed. We resolved disagreements on inclusion at all stages by discussion among the two reviewers. Excel sheet was used during the full-text screening and quality assessment phases where the reviewers note down their justification and other comments as a basis for discussion.

### Reading the studies, determining how studies are related and translating the studies (stage 3, 4 and 5)

We first used NVivo software (version 1.4) to facilitate close reading and extraction and comparison of “concepts” from each selected paper. Contextual information of all included papers was first extracted and all papers were read thoroughly by two reviewers YZ and DM. The concept of “first-order constructs” (participants’ “common-sense interpretation in their own words”) and “second-order constructs” (researchers’ interpretation based on first-order constructs) is usually distinguished for meta-ethnography and usually “second-order constructs” are “data” for meta-ethnography reviews (22,23). In our synthesis, as some studies did not primarily focus on linking internship experience and career outcomes but have relevant quotations and data establishing that link, we decided to extract both “first-order constructs” and “second-order constructs” and use both to develop “third-order constructs” (reviewers’ interpretation of original authors’ interpretation).

Four “key” papers were selected based on their richness and career outcomes examined, to be read and examined by YZ and DM independently to identify and extract concepts from each paper, and then they discussed the concepts and compared any differences in their interpretation. The rest of the papers were extracted by YZ only but reviewed by DM. Concepts from all included papers were then clustered into relevant categories through constant discussion and comparison between YZ and DM. For example, “competition between peers”, “gathering information from peers”, “norms among peers” and “support from peers” were merged into “relationship with peers” at this step.

We then went back to the primary papers, “translated” and compared our newly developed conceptual categories in all papers to ensure that no additional data were ignored. YZ read through all the papers again multiple times and wrote notes and selected quotes from each paper focusing on the finalized categories. This translation was conducted in Microsoft Word and reflected in the translation table (see appendix 3).

### Synthesizing translations and expressing the synthesis (stage 6 and 7)

After completing the stages above, we organized the categories into higher-level themes to generate a conceptual model to illustrate how the FY and internship experiences influenced medical doctors’ career intention/decisions. This is different from other types of qualitative synthesis that stop analysis at the stage where they have theoretically saturated categories. There are three ways to synthesize translation for meta-ethnography, i.e. refutational synthesis, reciprocal synthesis and line-of-argument.

We decided to go with the “line-of-argument” approach which refers to linking the themes and categories together and making “a whole into something more than the parts alone”, using a storyline and conceptual model. The model and the categories were shared and discussed within the research team for validation and feedback.

We also applied the GRADE-CERQual framework to define our confidence at the “category” level. GRADE-CERQual includes four domains (methodological limitations, relevance, adequacy of data, coherence) and an overall rating of confidence (high, moderate, low, very low). The assessments were made by YZ and further discussed with DM.

### Search strategy

Qualitative filter: The InterTASC Information Specialists’ Sub-Group Search Filter Resource.

available at [www.york.ac.uk/inst/crd/intertasc/](http://www.york.ac.uk/inst/crd/intertasc/).

Database(s): Embase 1974 to present
Search Strategy:

| **#** | **Searches** | **Results** |
| --- | --- | --- |
| 1 | exp education, medical, graduate/ | 329433 |
| 2 | Clinical Clerkship/ | 14197 |
| 3 | (foundation adj2 doctor*).ti,ab. | 738 |
| 4 | "junior doctor*".ti,ab. | 5312 |
| 5 | "house officer*".ti,ab. | 2304 |
| 6 | (medical and (student* or trainee*)).ti,ab. | 108992 |
| 7 | (medical and (intern or interns or internship*)).ti,ab. | 5938 |
| 8 | "medical resident*".ti,ab. | 2512 |
| 9 | "medical officer*".ti,ab. | 4041 |
| 10 | 1 or 2 or 3 or 4 or 5 or 6 or 7 or 8 or 9 | 398231 |
| 11 | exp career choice/ | 399417 |
| 12 | (career and (pathway or development or support or planning or framework or advice or choice or information or decision)).ti,ab. | 22068 |
| 13 | ((specialty or specialism or speciality) and (choice or decision)).ti,ab. | 6282 |
| 14 | (migrat* or emigrat* or retention or recruit*).ti,ab. | 1225052 |
| 15 | (rural or remote or maldistribution or mal-distribution).ti,ab. | 262192 |
| 16 | ("public sector" or "private sector" or "dual practice" or "dual job" or "moonlight*").ti,ab. | 18623 |
| 17 | 11 or 12 or 13 or 14 or 15 or 16 | 1885228 |
| 18 | (("semi-structured" or semistructured or unstructured or informal or "in-depth" or indepth or "face-to-face" or structured or guide) adj3 (interview* or discussion* or questionnaire*)).ti,ab. or (focus group* or qualitative or ethnograph* or fieldwork or "field work" or "key informant").tw,kw. or qualitative research/ | 489542 |
| 19 | 10 and 17 and 18 | 4732 |
| 20 | limit 19 to (english language and yr="2000 - 2020") | 4351 |

*Qualitative filter used for Ovid Embase: Canadian Health Libraries Association* [*https://extranet.santecom.qc.ca/wiki/!biblio3s/doku.php?id=concepts:recherche-qualitative*](https://extranet.santecom.qc.ca/wiki/!biblio3s/doku.php?id=concepts:recherche-qualitative)

Database(s): Medline (Ovid MEDLINE® Epub Ahead of Print, In-Process & Other Non-Indexed Citations, Ovid MEDLINE® Daily and Ovid MEDLINE®) 1946 to present
Search Strategy:

| **#** | **Searches** | **Results** |
| --- | --- | --- |
| 1 | exp education, medical, graduate/ | 73159 |
| 2 | Clinical Clerkship/ | 5319 |
| 3 | (foundation adj2 doctor*).ti,ab. | 341 |
| 4 | "junior doctor*".ti,ab. | 3202 |
| 5 | "house officer*".ti,ab. | 1825 |
| 6 | (medical and (student* or trainee*)).ti,ab. | 77147 |
| 7 | (medical and (intern or interns or internship*)).ti,ab. | 3842 |
| 8 | "medical resident*".ti,ab. | 1720 |
| 9 | "medical officer*".ti,ab. | 3658 |
| 10 | 1 or 2 or 3 or 4 or 5 or 6 or 7 or 8 or 9 | 151559 |
| 11 | exp career choice/ | 23931 |
| 12 | (career and (pathway or development or support or planning or framework or advice or choice or information or decision)).ti,ab. | 17168 |
| 13 | ((specialty or specialism or speciality) and (choice or decision)).ti,ab. | 4283 |
| 14 | (migrat* or emigrat* or retention or recruit*).ti,ab. | 910858 |
| 15 | (rural or remote or maldistribution or mal-distribution).ti,ab. | 218779 |
| 16 | ("public sector" or "private sector" or "dual practice" or "dual job" or "moonlight*").ti,ab. | 15580 |
| 17 | 11 or 12 or 13 or 14 or 15 or 16 | 1163766 |
| 18 | ((("semi-structured" or semistructured or unstructured or informal or "in-depth" or indepth or "face-to-face" or structured or guide) adj2 (interview* or discussion* or questionnaire*)) or (focus group* or qualitative or ethnograph* or fieldwork or "field work" or "key informant")).tw,kw. or interviews as topic/ or focus groups/ or narration/ or qualitative research/ | 423340 |
| 19 | 10 and 17 and 18 | 2001 |
| 20 | limit 19 to (english language and yr="2000 - 2020") | 1754 |

*Qualitative filter used for Ovid Medline: Canadian Health Libraries Association* [*https://extranet.santecom.qc.ca/wiki/!biblio3s/doku.php?id=concepts:recherche-qualitative*](https://extranet.santecom.qc.ca/wiki/!biblio3s/doku.php?id=concepts:recherche-qualitative)

Database(s): **Global Health**1973 to 2021 Week 21
Search Strategy:

| **#** | **Searches** | **Results** |
| --- | --- | --- |
| 1 | (medical education or medical students).sh. | 6731 |
| 2 | clinical experience.sh. | 107 |
| 3 | (foundation adj2 doctor*).ti,ab. | 12 |
| 4 | "junior doctor*".ti,ab. | 196 |
| 5 | "house officer*".ti,ab. | 119 |
| 6 | (medical and (student* or trainee*)).ti,ab. | 9742 |
| 7 | (medical and (intern or interns or internship*)).ti,ab. | 535 |
| 8 | "medical resident*".ti,ab. | 187 |
| 9 | "medical officer*".ti,ab. | 917 |
| 10 | 1 or 2 or 3 or 4 or 5 or 6 or 7 or 8 or 9 | 13520 |
| 11 | career choice/ | 211 |
| 12 | (career and (pathway or development or support or planning or framework or advice or choice or information or decision)).ti,ab. | 1626 |
| 13 | ((specialty or specialism or speciality) and (choice or decision)).ti,ab. | 301 |
| 14 | (migrat* or emigrat* or retention or recruit*).ti,ab. | 128402 |
| 15 | (rural or remote or maldistribution or mal-distribution).ti,ab. | 100789 |
| 16 | ("public sector" or "private sector" or "dual practice" or "dual job" or "moonlight*").ti,ab. | 6671 |
| 17 | 11 or 12 or 13 or 14 or 15 or 16 | 229698 |
| 18 | (("semi-structured" or semistructured or unstructured or informal or "in-depth" or indepth or "face-to-face" or structured or guide) adj3 (interview* or discussion* or questionnaire*)).ti,ab. or (focus group* or qualitative or ethnograph* or fieldwork or "field work" or "key informant").ti,ab,hw. or qualitative research/ | 97617 |
| 19 | 10 and 17 and 18 | 400 |
| 20 | limit 19 to (english language and yr="2000 - 2020") | 365 |

*Qualitative filter used for Ovid Global Health: modified from Ovid Embase, changed “tw,kw.” to “ti,ab,hw”*

Database(s): PsycINFO 1806 to present
Search Strategy:

| **#** | **Searches** | **Results** |
| --- | --- | --- |
| 1 | exp Medical Education/ or exp Medical Students/ | 31001 |
| 2 | exp Medical Internship/ or exp Medical Residency/ | 5111 |
| 3 | (foundation adj2 doctor*).ti,ab. | 52 |
| 4 | "junior doctor*".ti,ab. | 438 |
| 5 | "house officer*".ti,ab. | 304 |
| 6 | (medical and (student* or trainee*)).ti,ab. | 24791 |
| 7 | (medical and (intern or interns or internship*)).ti,ab. | 934 |
| 8 | "medical resident*".ti,ab. | 547 |
| 9 | "medical officer*".ti,ab. | 444 |
| 10 | 1 or 2 or 3 or 4 or 5 or 6 or 7 or 8 or 9 | 42708 |
| 11 | exp Occupational Choice/ | 8975 |
| 12 | (career and (pathway or development or support or planning or framework or advice or choice or information or decision)).ti,ab. | 31145 |
| 13 | ((specialty or specialism or speciality) and (choice or decision)).ti,ab. | 1091 |
| 14 | (migrat* or emigrat* or retention or recruit*).ti,ab. | 165607 |
| 15 | (rural or remote or maldistribution or mal-distribution).ti,ab. | 55763 |
| 16 | ("public sector" or "private sector" or "dual practice" or "dual job" or "moonlight*").ti,ab. | 8212 |
| 17 | 11 or 12 or 13 or 14 or 15 or 16 | 258048 |
| 18 | (((("semi-structured" or semistructured or unstructured or informal or "in-depth" or indepth or "face-to-face" or structured or guide or guides) adj3 (interview* or discussion* or questionnaire*)) or (focus group* or qualitative or ethnograph* or fieldwork or "field work" or "key informant")).ti,ab,id. or exp qualitative research/ or exp interviews/ or exp group discussion/ or qualitative study.md.) not "Literature Review".md. | 412023 |
| 19 | 10 and 17 and 18 | 809 |
| 20 | limit 19 to (english language and yr="2000 - 2020") | 767 |

*Qualitative filter used for Ovid PsycINFO: University of Texas https://libguides.sph.uth.tmc.edu/search_filters/ovid_psycinfo_filters*

Database: Web of Science - Science Citation Index Expanded (SCI-EXPANDED) --1900-present & Social Sciences Citation Index (SSCI) --1900-present

- (TS=( "foundation doctor*" OR "junior doctor*" OR "house officer*" OR "medical student*" OR "medical trainee*" OR "medical intern" OR "medical interns" OR "medical internship" OR "medical internships" OR "medical resident*" OR "medical officer*" ) )  AND LANGUAGE: (English)
- (TS=( "career" OR "specialty" OR "specialism" OR "speciality" OR "migrat*" OR "emigrat*" OR "remote" OR "rural" OR "maldistribution" OR "mal-distribution" OR "public sector" OR "private sector" OR "dual practice" OR "dual job" OR "moonlight*" ) )  AND LANGUAGE: (English)
- (TS = ( "semi-structured" OR "semistructured" OR "unstructured" OR "informal" OR "in-depth" OR "indepth" OR "face-to-face" OR "structured" OR "guide" OR "guides") )  AND LANGUAGE: (English)
- (TS = ("interview*" OR "discussion*" OR "questionnaire*" OR "focus group" OR "focus groups" OR "qualitative" OR "ethnograph*" OR "fieldwork" OR "field work" OR "key informant") )  AND LANGUAGE: (English)


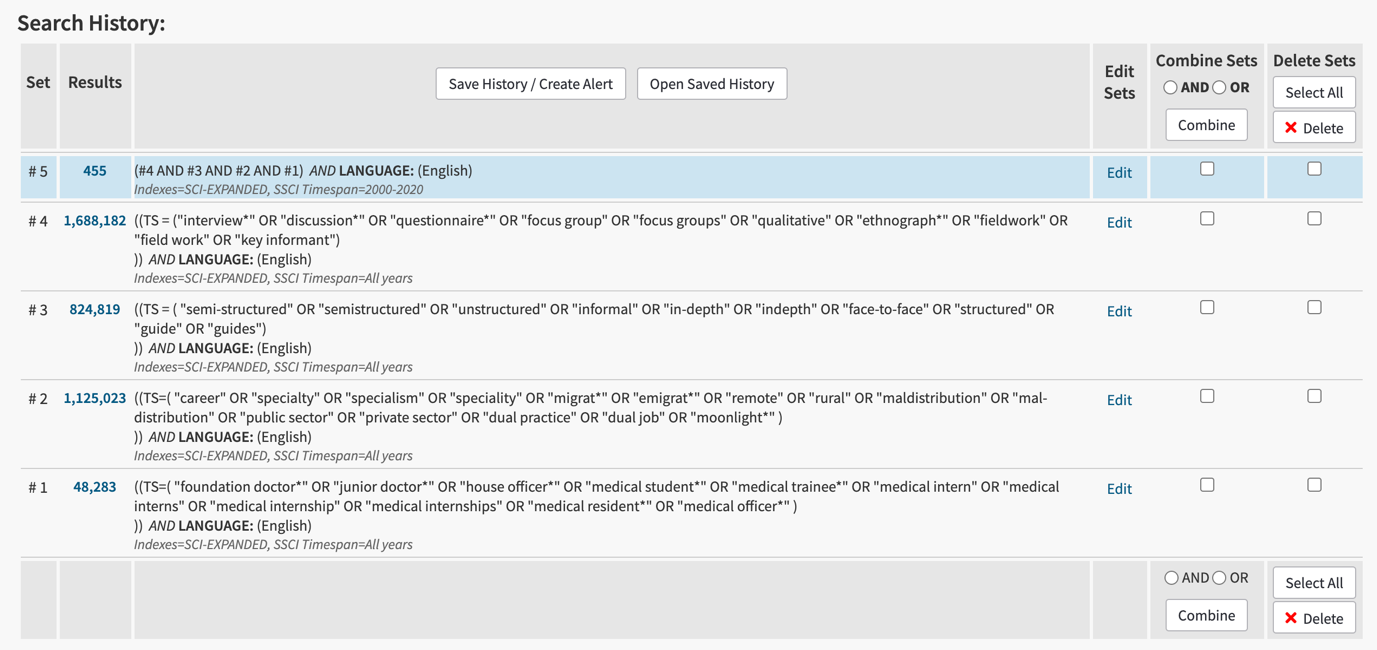


*Qualitative filter used for Ovid Global Health: modified from Canadian Health Libraries Association PubMed filter* [*https://extranet.santecom.qc.ca/wiki/!biblio3s/doku.php?id=concepts:recherche-qualitative*](https://extranet.santecom.qc.ca/wiki/!biblio3s/doku.php?id=concepts:recherche-qualitative)

### Papers excluded at full-text stage

| ID | title | journal | Reason for exclusion |
| --- | --- | --- | --- |
| 1 | '[It's] more than just medicine': The value and sustainability of mandatory, non-clinical, short-term rural placements in a Western Australian medical school | Medical teacher | not internship |
| 2 | 'Doctors ready to be posted are jobless on the street...' the deployment process and shortage of doctors in Tanzania | Human resources for health | no clear link between internship experience and career decision |
| 3 | 'If you can't make it, you're not tough enough to do medicine': a qualitative study of Sydney-based medical students' experiences of bullying and harassment in clinical settings | Bmc Medical Education | no clear link between internship experience and career decision |
| 5 | A decade of Australian Rural Clinical School graduates--where are they and why? | Rural and remote health | not internship |
| 6 | A framework for developing rural academic general practices: a qualitative case study in rural Victoria | Rural and remote health | no clear link between internship experience and career decision |
| 7 | A qualitative exploratory study: Using medical students' experiences to review the role of a rural clinical attachment in KwaZulu-Natal | South African Family Practice | not internship |
| 8 | A qualitative inquiry into the challenges of medical education for retention of general practitioners in rural and underserved areas of Iran | Journal of Preventive Medicine and Public Health | not internship |
| 9 | A Qualitative Investigation of the Experiences of Students and Preceptors Taking Part in Remote and Rural Community Experiential Placements During Early Medical Training | Journal of medical education and curricular development | not internship |
| 10 | A Qualitative Study Exploring the Determinants, Coping, and Effects of Stress in United Kingdom Trainee Doctors | Academic psychiatry : the journal of the American Association of Directors of Psychiatric Residency Training and the Association for Academic Psychiatry | no clear link between internship experience and career decision |
| 11 | A qualitative study of factors influencing under-represented medical students' Decision making about residency training programs | Journal of General Internal Medicine | abstract only |
| 12 | A qualitative study of medical students' attitudes to careers in general practice | Education for Primary Care | not internship |
| 13 | A qualitative study on factors that influence Turkish medical students' decisions to become family physicians after the health transformation Programme | North American Journal of Medical Sciences | not internship |
| 14 | A required rural health module increases students' interest in rural health careers | Rural and Remote Health | not internship |
| 15 | A Surgeon Led Clinically Focused Anatomy Course Increases Student Selection of General Surgery As a Career | Journal of Surgical Education | not internship |
| 17 | A taste of rural Highlands and Islands general practice: evaluation of remote placements in GP specialty training | Education for primary care : an official publication of the Association of Course Organisers, National Association of GP Tutors, World Organisation of Family Doctors | not internship |
| 18 | Academic career in medicine: requirements and conditions for successful advancement in Switzerland | BMC health services research | focus not on internship experience |
| 19 | An exploration of when urban background medical students become interested in rural practice | Rural and remote health | focus not on internship experience |
| 20 | Areas of distinction - A chance to try out an academic or leadership career during internal medicine residency training | Journal of General Internal Medicine | abstract only |
| 22 | Attracting diverse talent to academia: Perspectives of medical students and residents | Journal of Career Development | not internship |
| 24 | Attracting neurology's next generation: A qualitative study of specialty choice and perceptions | Neurology | no clear link between internship experience and career decision |
| 25 | Attrition from surgical residency training: Perspectives from those who left | American Journal of Surgery | not internship |
| 26 | Australian medical students' intentions in relation to practice location: their short- and long-term time frame | Australian Journal of Rural Health | short report only |
| 27 | Back in the day. What are surgeon bloggers saying about their careers? | Journal of Surgical Education | focus not on internship experience |
| 28 | Balancing Two Cultures: American Indian/Alaska Native Medical Students' Perceptions of Academic Medicine Careers | Journal of community health | not internship |
| 29 | Barriers and facilitating factors in the professional careers of international medical graduates | Medical education | no clear link between internship experience and career decision |
| 30 | Barriers facing junior doctors in rural practice | Rural and remote health | no clear link between internship experience and career decision |
| 32 | Becoming a GP: a qualitative study of the career interests of medical students | Australian family physician | no clear link between internship experience and career decision |
| 33 | Becoming an academic doctor: perceptions of scholarly careers | Medical education | no clear link between internship experience and career decision |
| 34 | Being the 'med reg': An exploration of junior doctors' perceptions of the medical registrar role | Journal of the Royal College of Physicians of Edinburgh | no clear link between internship experience and career decision |
| 35 | Beyond recruitment: A model program for strengthening retention of under-represented minority pediatric trainees in academia | Academic Pediatrics | abstract only |
| 36 | Building a local medical workforce in Tasmania: where are international fee-paying medical graduates likely to work? | Rural and remote health | not internship |
| 37 | Building health system capacity through medical education: A targeted needs assessment to guide development of a structured internal medicine curriculum for medical interns in Botswana | Annals of Global Health | no clear link between internship experience and career decision |
| 38 | Can a family medicine rotation improve medical students' knowledge, skills and attitude towards primary care in Vietnam? a pre-test - post-test comparison and qualitative survey | Tropical Medicine and International Health | not internship |
| 39 | Can i cut it? Medical students' perceptions of surgeons and surgical careers | American Journal of Surgery | not internship |
| 40 | Career choices and what influences Nepali medical students and young doctors: a cross-sectional study | Human Resources for Health | not internship |
| 41 | Career choices: Recent trends in health professions education among doctors | Pakistan Journal of Medical and Health Sciences | no clear link between internship experience and career decision |
| 42 | Career decision making in undergraduate medical education | Canadian medical education journal | not internship |
| 43 | Career destination and reason for career destination preferences among medical graduates from Christian Medical College Vellore - does rural service obligation increase retention of medical graduates in rural service? | Journal of Family Medicine and Primary Care | survey |
| 44 | Change of place, change of pace, change of status: rural community training for junior doctors, does it influence choices of training and career? | Rural and Remote Health | not internship |
| 45 | Choosing a career in medicine: The motivations of medical students from the University of Cape Town | Education for Primary Care | not internship |
| 46 | Choosing child and adolescent psychiatry: Factors influencing medical students | Journal of the Canadian Academy of Child and Adolescent Psychiatry | Not clear connection with internship experience and career decision |
| 47 | Choosing family medicine - What influences medical students? | Canadian Family Physician | no clear link between internship experience and career decision |
| 48 | Community-based education: The influence of role modeling on career choice and practice location | Medical teacher | survey |
| 49 | Determinants of first practice location: Among Manitoba medical graduates | Canadian Family Physician | survey |
| 50 | Doctors becoming GPs: GP registrars' experience of medical training and motivations for going into general practice | Education for Primary Care | focus not on internship experience |
| 51 | Doctors on the move: A qualitative study on the driving factors in a group of Egyptian physicians migrating to Germany | Globalization and Health | focus not on internship experience |
| 53 | Effects of compulsory rural vocational training for Australian general practitioners: A qualitative study | Australian Health Review | not internship |
| 54 | Emergency medicine as a career choice: What influences medical students throughout their schooling? | Canadian Journal of Emergency Medicine | abstract only |
| 55 | Encouragers and discouragers affecting medical graduates' choice of regional and rural practice locations | Rural and Remote Health | not internship |
| 56 | Enhancing the choice of general practice as a career | Australian Family Physician | focus not on internship experience |
| 58 | Evaluating the rural health placements of the Rural Support Network at the Faculty of Health Sciences, University of Cape Town | South African Family Practice | not internship |
| 59 | Examining intention among future physicians to practice in high need health professional shortage areas in Tennessee | Dissertation Abstracts International: Section B: The Sciences and Engineering | survey |
| 60 | Examining Medical Student Specialty Choice Through a Gender Lens: An Orientational Qualitative Study | Teaching and learning in medicine | not internship |
| 61 | Exploring Residents' Experience of Career Development Scholarship Tracks: A Qualitative Case Study Using Social Cognitive Career Theory | Teaching and learning in medicine | not internship |
| 62 | Exploring the career maze: An investigation of career intentions of medical graduates in Pakistan: A qualitative study | Pakistan Journal of Medical Sciences | focus not on internship experience |
| 63 | Extending a Conceptual Framework for Junior Doctors' Career Decision Making and Rural Careers: Explorers versus Planners and Finding the 'Right Fit' | International journal of environmental research and public health | focus not on internship experience |
| 64 | Factors affecting career preferences of medical students at the College of Medicine, Malawi | South African medical journal = Suid-Afrikaanse tydskrif vir geneeskunde | not internship |
| 65 | Factors affecting medical students' interests in working in rural areas in North India-A qualitative inquiry | PloS one | focus not on internship experience |
| 67 | Factors affecting the rural retention of medical graduates in lower northern Thailand | Journal of the Medical Association of Thailand | no clear link between internship experience and career decision |
| 68 | Factors Associated With Osteopathic Primary Care Residency Choice Decisions | The Journal of the American Osteopathic Association | not internship |
| 69 | Factors behind job preferences of Peruvian medical, nursing and midwifery students: a qualitative study focused on rural deployment | Human Resources for Health | not internship |
| 70 | Factors considered by medical students when formulating their specialty preferences in Japan: findings from a qualitative study | BMC medical education | not internship |
| 71 | Factors impacting the solo remote placement experiences of undergraduate James Cook University medical students: A mixed-methods pilot study | The Australian journal of rural health | focus not on internship experience |
| 72 | Factors Influencing Medical Students' Choice For Family Medicine As A Specialty In Pakistan | Journal of Ayub Medical College, Abbottabad : JAMC | no clear link between internship experience and career decision |
| 73 | Factors influencing recruitment and retention of foundation doctors in geographically unpopular locations | Future hospital journal | focus not on internship experience |
| 74 | Factors influencing the choice of anesthesia as a career in a developing country | Middle East Journal of Anesthesiology | survey |
| 75 | Factors influencing trainee doctor emigration in a high income country: a mixed methods study | Human resources for health | focus not on internship experience |
| 76 | Factors that influence a career choice in primary care: A mixed-methods study among medical students starting the social service program in Honduras | Dissertation Abstracts International: Section B: The Sciences and Engineering | focus not on internship experience |
| 77 | Factors that influence radiographers' decisions to pursue postgraduate education: An exploratory qualitative study | Journal of Medical Imaging and Radiation Sciences | population not doctor |
| 78 | Family medicine as a career option: How students' attitudes changed during medical school | Canadian Family Physician | survey |
| 80 | Foundation year one trainees feel simulation training will enhance learning of acute medicine, and development of early confidence in acute training may impact upon long term career choice | Medical Education, Supplement | abstract only |
| 81 | Gender and surgical careers: How can we better support women in surgery? | Medical Education, Supplement | abstract only |
| 82 | GP recruitment and retention: a qualitative analysis of doctors' comments about training for and working in general practice | Occasional paper (Royal College of General Practitioners) | survey |
| 85 | Identifying characteristics that students, interns and residents look for in their role models | Medical education | survey |
| 86 | Identifying motivations and personality of rural doctors: A study in Nusa Tenggara Timur, Indonesia | Education for health (Abingdon, England) | no clear link between internship experience and career decision |
| 87 | Identifying the motivators to engage african american students into careers in public health | Dissertation Abstracts International: Section B: The Sciences and Engineering | population not doctor |
| 90 | Influences on students' career decisions concerning general practice: A focus group study | British Journal of General Practice | not internship |
| 91 | Institutional conditions and individual experiences in the career-entry period of Swiss medical residents - A qualitative study | Swiss Medical Weekly | survey |
| 92 | Intellectual stimulation in family medicine: An international qualitative study of student perceptions | BJGP Open | not internship |
| 93 | Investigating a rural rotation in the Mississippi Delta utilizing reflective writings: A qualitative study | Dissertation Abstracts International Section A: Humanities and Social Sciences | not internship |
| 94 | Job satisfaction of rural medical interns: A qualitative study | The Australian journal of rural health | no clear link between internship experience and career decision |
| 96 | Junior doctors' views of how their undergraduate clinical electives in palliative care influenced their current practice of medicine | Academic medicine : journal of the Association of American Medical Colleges | focus not on internship experience |
| 97 | Longitudinal Integrated Foundation Training: uplifting perspectives | Medical education | abstract only |
| 99 | Medical brain drain in Uganda: Causes and potential remedies | Annals of Global Health | abstract only |
| 100 | Medical migration: A qualitative exploration of the atypical path of Japanese international medical graduates | Medical teacher | focus not on internship experience |
| 101 | Medical student and psychiatrist perceptions towards a psychiatric career | Mental Health Review Journal | population not doctor |
| 102 | Medical Student Decision Making Regarding Pursuit of a Public Health Degree | Family medicine | not internship |
| 103 | Medical student experiences in prison health services and social cognitive career choice: a qualitative study | BMC medical education | not internship |
| 104 | Medical student observations on a career in psychiatry | Australian and New Zealand Journal of Psychiatry | survey |
| 105 | Medical student's perceptions of a career in gastroenterology: A peer led study | Gut | abstract only |
| 106 | Medical students and rural general practitioners: congruent views on the reality of recruitment into rural medicine | Australian Journal of Rural Health | not internship |
| 107 | Medical students on long-term rural clinical placements and their perceptions of urban and rural internships: a qualitative study | BMC medical education | not internship |
| 108 | Medical students' attitudes towards careers in primary care in Singapore | BMC medical education | not internship |
| 109 | Medical students' perceptions of general practice as a career; a phenomenological study using socialisation theory | Education for primary care : an official publication of the Association of Course Organisers, National Association of GP Tutors, World Organisation of Family Doctors | not internship |
| 110 | Medical students' perceptions of primary care: The influence of tutors, peers and the curriculum | Education for Primary Care | not internship |
| 112 | Overseas-trained doctors in Australia: Community integration and their intention to stay in a rural community | Australian Journal of Rural Health | no clear link between internship experience and career decision |
| 113 | Perceptions of graduates from Africa's first emergency medicine training program at the University of Cape Town/Stellenbosch University | CJEM | no clear link between internship experience and career decision |
| 114 | Perceptions of Malaysian medical students from different academic years on primary care: a qualitative research | Family medicine and community health | not internship |
| 115 | Postgraduate career intentions of medical students and recent graduates in Malawi: a qualitative interview study | BMC medical education | not internship |
| 116 | Postgraduates' perceptions of preparedness for work as a doctor and making future career decisions: support for rural, non-traditional medical schools | Education for health (Abingdon, England) | no clear link between internship experience and career decision |
| 117 | Pre-registration house officers' comments on working in the NHS: a qualitative study of the views of UK medical graduates of 1999 | Medical teacher | survey |
| 119 | Preferences of doctors for working in rural Islamabad Capital Territory, Pakistan: a qualitative study | Journal of Ayub Medical College | not internship |
| 120 | Prevocational Integrated Extended Rural Clinical Experience (PIERCE): cutting through the barriers to prevocational rural medical education | Rural and remote health | not internship |
| 121 | Pursing a career in academic surgery among African American medical students | American journal of surgery | no clear link between internship experience and career decision |
| 122 | Push and stay factors affecting Irish medical student migration intentions | Irish journal of medical science | survey |
| 123 | Qualitative study of medical students' experiences of a psychiatric attachment | Psychiatrist | not internship |
| 124 | Raising concerns in the current NHS climate: a qualitative study exploring junior doctors' attitudes to training and teaching | Future healthcare journal | no clear link between internship experience and career decision |
| 126 | Review of final-year medical students' rural attachment at district hospitals in KwaZulu-Natal: Student perspectives | South African Family Practice | survey |
| 127 | Role models play the greatest role - a qualitative study on reasons for choosing postgraduate training at a university hospital | GMS Zeitschrift fur medizinische Ausbildung | no clear link between internship experience and career decision |
| 128 | Rural exposure during medical education and student preference for future practice location - a case of Botswana | African Journal of Primary Health Care and Family Medicine | not internship |
| 130 | Rural rotations for interns: a demonstration programme in South Australia | The Australian journal of rural health | no clear link between internship experience and career decision |
| 132 | Southeast and East Asian American medical students' perceptions of careers in academic medicine | Journal of Career Development | no clear link between internship experience and career decision |
| 134 | Specialization training in Malawi: a qualitative study on the perspectives of medical students graduating from the University of Malawi College of Medicine | BMC medical education | not internship |
| 135 | Specialty income and career decision making: a qualitative study of medical student perceptions | Medical education | not internship |
| 136 | Specialty preferences of 1(st) year medical students in a Saudi Medical School - Factors affecting these choices and the influence of gender | Avicenna journal of medicine | survey |
| 137 | Stability and Change in the Journeys of Medical Trainees: A 9-Year, Longitudinal Qualitative Study | Academic medicine : journal of the Association of American Medical Colleges | no clear link between internship experience and career decision |
| 138 | Stick or twist? Career decision-making during contractual uncertainty for NHS junior doctors | BMJ open | no clear link between internship experience and career decision |
| 139 | Stories from early-career women physicians who have left academic medicine: A qualitative study at a single institution | Academic Medicine | no clear link between internship experience and career decision |
| 140 | Students' perspectives on the fourth year of medical school: A mixed-methods analysis | Academic Medicine | not internship |
| 141 | Support for rural practice: female physicians and the life-career interface | Rural and remote health | no clear link between internship experience and career decision |
| 142 | Taking a break: doctors opt out of training after foundation year 2 | BMJ (Online) | short report only |
| 143 | The career aspirations and location intentions of James Cook University's first cohort of medical students: a longitudinal study at course entry and graduation | Rural and remote health | survey |
| 145 | The hidden curriculum of the medical care for elderly patients in medical education: a qualitative study | Gerontology & geriatrics education | not internship |
| 147 | The impact of interest: How do family medicine interest groups influence medical students? | Canadian Family Physician | no clear link between internship experience and career decision |
| 148 | The impact of the internal medicine sub-internship on medical student career choice | Journal of General Internal Medicine | not internship |
| 149 | The Influence of Academic Discourses on Medical Students' Identification With the Discipline of Family Medicine | Academic Medicine | no clear link between internship experience and career decision |
| 150 | The learner's perspective in GP teaching practices with multi-level learners: a qualitative study | BMC medical education | no clear link between internship experience and career decision |
| 151 | The lived experience of stress in British South-Asian medical students and junior doctors | Work (Reading, Mass.) | no clear link between internship experience and career decision |
| 153 | The parallel rural community curriculum: an integrated clinical curriculum based in rural general practice | Medical education | no clear link between internship experience and career decision |
| 154 | The perceived usefulness of community based education and service (COBES) regarding students' rural workplace choices | Bmc Medical Education | not internship |
| 155 | The role of gender in the decision to pursue a surgical career: A qualitative, interview-based study | Canadian medical education journal | no clear link between internship experience and career decision |
| 157 | Trainee attrition in obstetrics and gynaecology training - A qualitative analysis of trainee attitudes | BJOG: An International Journal of Obstetrics and Gynaecology | abstract only |
| 158 | UK medical students' attitudes towards their future careers and general practice: a cross-sectional survey and qualitative analysis of an Oxford cohort | BMC medical education | survey |
| 159 | Understanding rural clinical learning spaces: Being and becoming a doctor | Medical teacher | no clear link between internship experience and career decision |
| 160 | Understanding the 'four directions of travel': qualitative research into the factors affecting recruitment and retention of doctors in rural Vietnam | Human Resources for Health | no clear link between internship experience and career decision |
| 162 | Views of UK doctors in training on the timing of choosing a clinical specialty: Quantitative and qualitative analysis of surveys 3 years after graduation | Postgraduate Medical Journal | survey |
| 163 | What are the barriers faced by under-represented minorities applying to dermatology? A qualitative cross-sectional study of applicants applying to a large dermatology residency program | Journal of the American Academy of Dermatology | no clear link between internship experience and career decision |
| 164 | What do doctors want? Incentives to increase rural recruitment and retention in India | BMC Proceedings | abstract only |
| 165 | What do medical students think about primary care in Malaysia? A qualitative study | Education for Primary Care | population include medical student |
| 166 | What factors in rural and remote extended clinical placements may contribute to preparedness for practice from the perspective of students and clinicians? | Medical teacher | no clear link between internship experience and career decision |
| 168 | What leads to the subjective perception of a 'rural area'? A qualitative study with undergraduate students and postgraduate trainees in Germany to tailor strategies against physician's shortage | Rural and remote health | no clear link between internship experience and career decision |
| 169 | What makes a medical student avoid or enter a career in urology? Results of an international survey | The Journal of urology | survey |
| 171 | Where are they working? a case study of twenty Cuban-trained South African doctors | African Journal of Primary Health Care and Family Medicine | no clear link between internship experience and career decision |
| 174 | Why are you draining your brain? Factors underlying decisions of graduating Lebanese medical students to migrate | Social Science & Medicine | no clear link between internship experience and career decision |
| 175 | Why choose psychiatry? Report on a qualitative workshop | The Psychiatrist | abstract only |
| 177 | Why do Danish junior doctors choose general practice as their future specialty? Results of a mixed-methods survey | European Journal of General Practice | survey |
| 178 | Why do doctors in Norway choose general practice and remain there? A qualitative study about motivational experiences | Scandinavian journal of primary health care | no clear link between internship experience and career decision |
| 179 | Why do junior doctors not want to work in a rural location, and what would induce them to do so? | The Australian journal of rural health | survey |
| 181 | Why doctors choose small towns: A developmental model of rural physician recruitment and retention | Social Science and Medicine | no clear link between internship experience and career decision |
| 182 | Why geriatrics? Academic geriatricians' perceptions of the positive, attractive aspects of geriatrics | Family Medicine | no clear link between internship experience and career decision |
| 183 | Why UK medical students change career preferences: an interview study | Perspectives on Medical Education | not internship |
| 184 | Why would I choose a career in family medicine?: Reflections of medical students at 3 universities | Canadian family physician Medecin de famille canadien | no clear link between internship experience and career decision |
| 185 | Women paediatricians: What made them choose their career? | Journal of Health, Organisation and Management | no clear link between internship experience and career decision |
| 186 | Women physicians: choosing a career in academic medicine | Academic medicine : journal of the Association of American Medical Colleges | no clear link between internship experience and career decision |
| 187 | Young surgeons' challenges at the start of their clinical residency: a semi-qualitative study | Innovative surgical sciences | no clear link between internship experience and career decision |
