# Supplementary material for: How do foundation year and internship experience shape doctors’ career intentions and decisions? A meta-ethnography
Source: Med Teach. Author manuscript; Available in PMC 2024 Jan 19. (PMC7615548; doi:10.1080/0142159X.2022.2106839)
Supplement: Appendix 2 [file EMS193419-supplement-Appendix_2.docx]

**Appendix 2. CASP and quality appraisal (including papers that are further excluded after the quality appraisal stage)**

| Author | Q1 Clear statement of aim | Q2 Method appropriate | Q3 Design appropriate | Q4 Recruitment strategy | Q5 Data collection | Q6 Relationship | Q7 Ethics | Q8 Data analysis | Q9 Finding clear | Q10 Valuable | Total score | Recommendation |
| --- | --- | --- | --- | --- | --- | --- | --- | --- | --- | --- | --- | --- |
| Alberti 2017 | 2 | 2 | 2 | 1 | 2 | 0 | 2 | 1 | 2 | 2 | 16 | satisfactory |
| Appleton 2017 | 2 | 2 | 2 | 2 | 2 | 2 | 2 | 2 | 2 | 2 | 20 | key |
| Beattie 2017 | 2 | 2 | 2 | 2 | 2 | 2 | 2 | 2 | 2 | 2 | 20 | satisfactory |
| Brodribb 2016 | 2 | 2 | 2 | 2 | 2 | 0 | 2 | 2 | 2 | 2 | 18 | satisfactory |
| Croghan 2020 | 2 | 2 | 2 | 2 | 2 | 1 | 1 | 1 | 1 | 2 | 16 | satisfactory |
| Cuesta-Briand 2020 | 2 | 2 | 2 | 2 | 2 | 0 | 1 | 2 | 2 | 2 | 17 | key |
| Cutajar 2020 | 2 | 1 | 1 | 2 | 1 | 2 | 0 | 0 | 1 | 1 | 11 | methodology fatally flawed |
| Elliott 2007 | 2 | 1 | 1 | 2 | 2 | 1 | 2 | 0 | 1 | 1 | 13 | methodology fatally flawed |
| Essa 2010 | 2 | 2 | 2 | 1 | 2 | 0 | 2 | 2 | 2 | 2 | 17 | key |
| Firth 2011 | 2 | 2 | 1 | 2 | 2 | 0 | 2 | 2 | 2 | 1 | 16 | satisfactory |
| Harris 2020 | 2 | 2 | 2 | 2 | 2 | 1 | 2 | 2 | 2 | 2 | 19 | key |
| Liang 2019 | 1 | 2 | 2 | 2 | 2 | 2 | 2 | 2 | 2 | 2 | 19 | satisfactory |
| Ludwig 2018 | 1 | 2 | 2 | 2 | 2 | 1 | 1 | 2 | 2 | 1 | 16 | satisfactory |
| Merrett 2017 | 2 | 2 | 1 | 2 | 2 | 0 | 2 | 1 | 2 | 2 | 16 | satisfactory |
| Olsson 2019 | 2 | 2 | 2 | 1 | 2 | 0 | 2 | 2 | 2 | 2 | 17 | key |
| Parker 2014 | 2 | 2 | 2 | 2 | 2 | 2 | 2 | 2 | 2 | 2 | 20 | key |
| Querido 2018 | 2 | 2 | 2 | 2 | 2 | 2 | 2 | 2 | 2 | 2 | 20 | satisfactory |
| Rizan 2019 | 2 | 2 | 2 | 2 | 2 | 0 | 2 | 2 | 2 | 2 | 18 | key |
| Scanlan 2018 | 2 | 2 | 2 | 2 | 2 | 2 | 2 | 2 | 2 | 2 | 20 | key |
| Sen Gupta 2008 | 1 | 1 | 1 | 1 | 1 | 0 | 1 | 0 | 1 | 1 | 8 | methodology fatally flawed |
| Smith 2018 | 2 | 2 | 2 | 2 | 2 | 2 | 2 | 2 | 2 | 2 | 20 | key |
| Spooner 2017 | 2 | 2 | 2 | 2 | 2 | 0 | 2 | 2 | 2 | 2 | 18 | key |
| Sreedaran 2018 | 2 | 2 | 2 | 1 | 1 | 2 | 2 | 2 | 1 | 1 | 16 | satisfactory |
| Van Hamel 2020 | 2 | 2 | 2 | 2 | 2 | 2 | 2 | 2 | 2 | 2 | 20 | key |
| Williams 2000 | 2 | 2 | 2 | 1 | 2 | 2 | 0 | 2 | 2 | 2 | 17 | key |
| Woodward 2018 | 2 | 1 | 1 | 2 | 2 | 0 | 2 | 1 | 2 | 1 | 14 | satisfactory |
