# Supplementary material for: How do foundation year and internship experience shape doctors’ career intentions and decisions? A meta-ethnography
Source: Med Teach. Author manuscript; Available in PMC 2024 Jan 19. (PMC7615548; doi:10.1080/0142159X.2022.2106839)
Supplement: Appendix 3 [file EMS193419-supplement-Appendix_3.docx]

**Appendix 3. Translation process**

1. **Finding which career option is “more me” – hands on experience, comparing between rotations and deciding on the best fit, both clinically and in general**

| **Reference** | **The “hand-on” experience and “real life” exposure** | **Positive experience, confidence and readiness** | **The workload, work-life balance, lifestyle** | **Well-being, emotional stress and the need to step off** | **Image of professions and self-identity** |
| --- | --- | --- | --- | --- | --- |
| Williams 2000  UK / PRHO / Specialty choice – surgery  CASP 17 (recruitment, ethics) / Key | - The amount of ward work often made it difficult for female PRHOs to get to theatre, however when they did manage to get to theatre they find the actual experience very enjoyable.   *“I sutured an appendicectomy last night, and it was terribly exciting and I love suturing. It was wonderful, and I loved giving this person a lovely scar. (PRHO 8)”* |  | - Working hours of surgeons being incompatible with hours bringing up a family   *“I like surgery but I know I definitely want a family, and I think to go into surgery it would mean delaying that for quite a while, and I don't know if I really want to. I mean, when you see the hours the consultants put in you just think, `Oh no, I just don't want to do that'. (PRHO 15)”*  *“I would like to stay in hospital surgery, but I'm a bit family oriented so I'm not sure… I want to have the nice house, 2-4 children, and be happily married and all that, and I'm not sure how that works when you're a female surgeon…(PRHO 12)”* |  | - There are desirable characteristics of potential surgeons including “push” and determination, surgery as “doing” rather than “thinking”, taking a narrow view of patients rather than holistically   *“Now I'm suddenly looking after someone's husband, someone's daughter, and it's quite stressful, because you don't want anything to happen to them because the same thing could happen to anyone in your family. So I don't know, that's not a very surgical opinion, that's a very medical thing to say, but I think it's important. I think it's sad that people think that it doesn't matter, the patient's just there but I do like to think about them, you do worry. (PRHO 12)”* |
| Scanlan 2018  UK / FY2 / General  CASP 20 / Key | - Direct experience and comparison with other rotations making interns realize what “fit” in terms of personality and what they enjoy.   *“Having the opportunity to do intensive care and anaesthetics as my first FY2 job which… because I was interested in it and I was given that by the programme director; he organised that, so I had experience in that before I applied, which I was very grateful for. having done that I knew that was what I wanted to do and so*  *I applied. (Story 2 Steven)”*  *“Anaesthetics is interesting but I didn’t think I want to do it. Didn’t get an A and E job but I got an intensive care job and then, I think, as I met the people with the emergency medicine doctors; I learned a bit more about how I liked to work…I think people talk about personalities and I thought maybe my personality wasn’t actually quite aligned to emergency medicine but I really enjoyed anaesthetics. (Story 2 Steven)”* | - Feeling confident after rotations on managing patients.   *“I felt much more confident, at the end of the four months, that I could assess, you know, intensive care patients and make basic decisions. Nothing fancy but at least hold the fort for like an hour or so. (Story 2 Steven)”* | - Hoping to be able to control working hours   *“Erm I think now, the longer I work for, not having any control over my annual leave, my days off, my time off, is really starting to really annoy me. So my next rota, for example, I have just been informed, you have three weeks’ annual leave, this is when they are, you can’t swap them, you can’t move them, tough. I think that that is a disgrace. the number of hours I have worked to be told, you’re not allowed to go on a family holiday that you’ve booked. You’re not allowed… I’ve been told I’m not allowed to go to a funeral before. I’ve been told I’m not allowed time off when I’m ill. That… I would have said, is the worst thing. I just, I don’t think it’s fair, and I think that bothers me more and more, I think, as time goes on. (Story 5 Clare)”* | - Feeling overwhelmed and exhausted in certain rotations   *“sometimes a bit overwhelming and exhausting… You’ve literally been at work, slept, and been at work for seven days; you’ve had no social interaction at all outside of work, and that also gets you down. Because, you know, you need to do, you see friends and do fun things that will let you relax, to be able to maintain a, kind of, healthy emotional state. (Story 4 Gemma)”* |  |
| Alberti 2017  UK / GPSpR / Specialty choice – GP  CASP 16 (recruitment, relationship, analysis) / Satisfactory | - Previous exposure to GP as FD positively influenced selecting GP as a career.   *“I think everyone should do a foundation rotation in GP, everybody. I think it will help not only people decide if they like it and what to do. But also having consultants understand what GPs actually do.”* |  |  |  | - Badmouthing of GP on social media, television and in newspaper, the general public’s lack of awareness what GP entails   *“Also everything in the press, not just now but over the last however many years, there is a lot in the press about GP’s and missing this missing that and misrepresentation and I think that as well does impact on people’s perception”* |
| Merrett 2017  UK / FY1 / Specialty choice – GP  CASP 16 (design, relationship, analysis) / Satisfactory | - More compelling and administrative issues in hospital clinicians when compared with GPs   *“I just can’t imagine myself running after secretaries and stuff for the rest of my life. I think it’s just the whole sort of packet, rules and regulations within hospitals, you can’t do certain things without certain people’s signatures, and you can’t do this without that.’ (Participant, group 1)”*   - Reluctance towards managing patients with mental health problems in GP, those perceived to be social rather than medical needs. Concern that might lose specific skill set   *“Every single patient that came in wasn’t really with a medical condition. It was more psychosocial, and so I can understand why a lot of my friends were, like: “I would never do this. Why have I spent 5 years learning all about the body, the anatomy, everything, and it’s my psychiatrist or psychologist role used?”’ (Participant, group 5)”*   - Concern about GPs as running a business rather than practicing medicine   *“‘If you were seeing patients all day, fine. But it’s the extra time — the 4 hours lunchtime that you’re not having lunch … it’s the other bits and pieces you’ve got, relating to QOF [Quality and Outcomes Framework] points, relating to managerial work, because general practice partners are also business people, really. It’s all that, and that’s not medicine.’ (Participant, group 3)”* |  | - Perceived quality of life being better in GP. However, the actual experience also makes interns realize GP is as stressful and work long hours as others   *“When I was working at [sic] GP, they actually worked really long hours, and the lifestyle didn’t seem that much better.’ (Participant, group 2)”* | - GPs being considered as a “lonely” specialty without support and team spirits   *“‘It’s a lot scarier, because you’re more on your own for a long time. Yeah, you might work in a general practice with other GPs, but you could just be working as a GP on your own, on an island somewhere, no one else to speak to, and that’s quite scary.’ (Participant, group 4)”*   - GPs increased litigation thus increased work stress and likelihood of burning out   *“Yeah. I’ve come across GPs that are going through, like, litigation processes, and have been absolutely burnt out with it. I think everyone at some point, whatever specialty, will miss a diagnosis. But you’re much more open to litigation in general practice.’ (Participant, group 3)”* | - Medical and patients’ perception and stigma of GP |
| Smith 2018  UK / FY1 / Migration  CASP 20 / Key | - F2 think the e-portfolio was cumbersome and too vague to be useful, and want to step off for a while,   *“If I can just not have to deal with any portfolio for two years I will be delighted. I think it just detracts so much from any enjoyment that you have… especially with the foundation e-portfolio because they have to design it so generically that some things that they want you to do can be incredibly difficult depending on the particular combination of jobs that you have. (P17)”* | - Not feeling skills had adequately prepared them to enter specialty training directly.   *“When I see people go and start their CMT [core medical training], they cover CCU [the coronary care unit] and renal on their nightshift. It's funny because at the moment I'm the one calling them about this abnormal ECG [electrocardiogram], but then you think, God, in four months’ time I'm the one who is getting called… that sounds awful. I think maybe I am just not quite ready to go into CMT yet. (P9)”* | - F2s have to stay late at work for jobs that are non-handoverable and get very busy   *“It’s things like ordering bloods for tomorrow. Things you know that should have been done during the day. But sometimes, you’re just so busy, it doesn’t get done. (P10)”*   - Poor work-life balance in the NHS and an expectation that there would be less work-life conflict abroad   *“I've spoken to people who are doing A&E there [in New Zealand], and it's very similar, but, less hours. So, they normally do four days a week, whereas, right now, I'm doing seven. I'm on seven days right now, and I'm working from either two or three, 'till midnight, every day. Next week, I'm going straight into nights. So that's two weeks that I cannot have any social life. In New Zealand, I can do the job for four days a week, and then have a nice weekend off. (P4)”*  *“‘She [another doctor] says the quality of life out there [in Australia] is so much better. She said when she worked here she was always stressed out, quite anxious as well… Then when she went out there she felt more supported and had enough time off to relax. She feels like she’s absolutely a completely different person because her work life now doesn’t affect her personal life. (P9)”* | - F2 feel like there were on a “conveyor belt” or “treadmill” of training and need to step off for a while or needing a break from medical training, from being a doctor, thinking about career progression, and hopefully could be more enthusiastic about work again.   *“You are on this conveyor belt, you're 17 and then you've signed up for life. I think, for me, it was just the last couple of years, you realise what you've actually committed to. If I commit to a training programme, that's six years or so and after that you are looking at trying to get consultancy posts quite quickly. I would think on that kind of timescale you are thinking about family and mortgages… I don't think you can really travel. This is an opportunity to go away for so long whilst still maintaining your clinical practice. (P6)”*  *“It seems that a lot of FY2’s that I know are quite burnt*  *out, to be honest. People are tired of, I don’t know if it’s tired of working, but… you get into medical school and then it’s five or six years and straight into foundation training and then you’re probably working towards entering medical school for many, many years before you enter medical school. So everyone just seems to need time off. (P12)”*  *“t’s also a break from having to think about your career too much, especially if you want to go into a competitive speciality. There can always be a pressure on you that it’s not just enough to do the day job -you have to get published, you have to do your audits, your posters, your presentations, there’s extra courses and working everything into your CV and just all that general stuff, whereas I think if you practice medicine abroad…well, I certainly don’t intend on pushing the seriously academic side of my CV too much when I just want to enjoy doing the job. (P17)”*  *“I feel like I should have a break for a year and probably that will make me feel more enthusiastic about working in the NHS again afterwards. (P1)”* |  |
| Spooner 2017  UK / FY2 / Specialty choice  CASP 18 (relationship) / Key | - Observing and assisting as medical student is significantly different to the experience of being a doctor being of different level of responsibility   *“I hated my job [psychiatry] but I loved it as a student… I just found it really depressing as a doctor whereas as a student I found all the stories really interesting. (GP0P18)”*  *“I don’t think you get the same experience as a medical student as you do as a junior doctor, no matter how much they try to, because you just don’t have that same responsibility. (GP2P12)”*  *“the realities of the job set in… everything’s great when you’re a student because you can just walk away an hour before the work’s done… when you’re dealing with it as a doctor you have to see things through to the end of the day and all the negative experiences and all the arguments with patients and relatives and all the complaints. (GP2P15)”* | - Consultants and team being supportive and help interns rebuild their damaged confidence   *“In my GP placement, had an amazing supervisor that was just really supportive and gave me feedback how it should have been given, and just kind of coaxed me through and built up my confidence again. (GP0P2)”*   - In other cases, losing confidence in certain areas, feeling uncomfortable in managing the intrinsic uncertainties of primary care leading to reluctance into choosing Gp   *“Even being in GP for those four months… dealing with, sort of, different, more chronic conditions and I really hated that I’d lost confidence, I’d forgotten things, I didn’t feel as confident with poorly patients. (GP0P10)”*   - Interns themselves finding opportunities to build confidence, using interpersonal skills, deal with variety and have access to supportive team which lead to positive experiences. | - Uncertainty if choosing GP then regular working hours would be achievable   “*With A&E you finish your shift and you go home, with GP you finish your surgery and then you do your clinic referrals and you do your letters and you do everything else and then eventually you get home and then you have other stuff to do and it just seems to take over your life. (GP0P18)”*   - Achieving good work-life balance, time for family and friends, exploring interest beyond medical work   *“I would love to do gastroenterology, but… I just know I wouldn’t have a good work-life balance. Work-life balance is really important to me, I’d probably say more so than what I want to do in my career… if I’m not enjoying myself out of work, it’s just not worth it for me. (GP0P10)”*   - Heavy workload in certain specialties and worry on patient safety   *“Paediatric registrars are incredible, they work phenomenally hard, they have horrendous hours… I don’t want to be doing that for the rest of my life, I can’t safely practice doing that for the rest of my life. (GP0P17)”* |  | - Status and respect related to certain specialties and how it related to specialty choices   *“I don’t care what my friends and family think, it’s the wider population. … I had the girl doing my nails one day. She said, ‘Oh, what kind of doctor are you going to be?’ I said, ‘I’m going to be a GP’. And she said, ‘Oh, do you have to go to medical school for that?’ And I just thought, … there’s just that a bit less respect, isn’t there, than, ‘Oh yes, I’m a brain surgeon. (GP1P16)”* |
| Ludwig 2018  Germany / Practice year / Specialty choice – GP  CASP 16 (statement, relationship, ethics, valuable) / Satisfactory | - Unexpected responsibility of being a GP requiring business knowledge, bureaucratic burden of practice and restriction on medical work but the healthcare system   *“Well, basically you end up running a medium-sized company, but never having learned anything of business admin [...], never anything about accounting, that’s shocking. (TN 5, Post, WB-, Rural-)”* | - Experience with diagnostic difficulty and using guidelines building confidence in treating patients.   *“[...] and using guidelines and evidence-based structures, you were able to gain confidence in dealing with patients. [...] you’re a doctor on your own and you can be a very good doctor.” (TN 8, Post, WB+, Rural+)”* | - Expected workload with GP but more flexible working hours, however sometimes fluctuating depending on the organization of practice structure.   *““Well, of course it’s a lot of time you end up investing, [...] But in return you also have the advantage of being free at lunch time, for example.” (TN 2 Post, WB+, Rural+)”*   - Whether proposed career is compatible with having a family.   *“I think, especially as a female GP, [...] it’s easier to manage having a family than, for example, as a senior physician in a clinic, with loads of weekend shifts, emergency shifts, being on-call etc. (TN 12, Post, WB-, Rural-)”*  *“And that’s usually the reason why it fails, ’cos the family has to tag along. [...] especially your partner, but also the kids [...] they got their school, their friends, making it less likely you’ll move. (TN 1, Post, WB+, Rural+)”* |  |  |
| Parker 2014  NZ / Final year undergrad / Specialty choice – GP  CASP 20 / Key |  | - Positive experience including being included, feeling welcomed and part of the team, having their own patient list, many opportunities for practicing. Negative experience was worse than no experience. |  |  |  |
| Olsson 2019  Sweden / Young doctor / Specialty choice  CASP 17 (recruitment, relationship) / Key |  |  |  |  | - Status symbol related to different specialty being reminded constantly   *“One is often reminded of that, that within the profession—although maybe it is actually only the case with other surgeons!—that surgery is a certain status symbol …///… which also comes from the fact that you are responsible…///…, you have a lot of power, and you should be aware of that when you talk to a patient before an operation, because you must give due respect to the fact that, in that position or relationship, the patient is very much in the opposite role, with no power at all. And they’re about to lie unconscious on the operating table, literally putting their life in your hands. And I maybe think that maybe contributes to what I certainly experience as the traditional view that surgeons are something extraordinary and powerful. Certainly, many surgeons consider themselves to be so (laughing). But that doesn’t mean that physicians are as important, but…’ (No. 15, surgery, woman)”*   - There is perceived status difference between specialties where surgery was the most prestigious and psychiatry and geriatrics are considered low status. Interns also have to defend their choices in front of others. |
| Querido 2018  Netherlands / Final transition year / General  CASP 20 / Key | - Experience during internship rotations, knowing what it contains and finding out which interns enjoy most.   *“Without an internship, you don’t know exactly what it contains. Yes, and when I started my rotations, that was the rotation, which I enjoyed most. Yeah, that is, hhhmm that is it.” (no.1, female)”* |  | - Choosing specialty based on the need for a work-life balance. Some also notice a work-life imbalance but are willing to accept it because other factors are more important.   *“… the work-life balance weighs for me. If you want children and a wife then part-time work is attractive. I would like to work hard, but for me it is important that at some time the working day ends. I want to finish at the end of the day and go home and not be called in again. That consideration influences my career preference at the moment. (no 17, male)”*  *“… I think about cardiology. It is sort of the only preference I have, although I also think of general practice as an option. Basically, I would like to focus on cardiology. … I think working hours will be heavy, but I do not think that is a reason to lose interest. It will be of big impact on my life, but I am willing to sacrifice that for it. (no. 24, female)”* |  |  |
| Brodribb 2016  Australia / Junior doctor / Rural practice  CASP 18 (relationship) / Satisfactory |  | - Negative experience with lack of professional support make interns feel not confident and anxiety-making   *“I ended up in a rural hospital with no support with very unwell people, which I didn’t feel conﬁdent managing. I had a couple of experiences with extremely sick patients and just me, or me and one other person...looking after them. It certainly put me off working there as a more senior person, because I found that just highly anxiety-making.*  *...So I ﬁnd it a bit terrifying that I was with one other person with people intubated and sedated, for ﬁve hours waiting for a helicopter to come and pick them up. I think that’s something that would...change my decision making process [about working in rural areas]. (F, 31, Registrar, Z)”* |  |  |  |
| Van Hamel 2020  UK / FY2 / Not going into specialty training  CASP 20 / Key |  | - “Too early” to decide on a specific career path, lack of flexibility within training schemes, difficulty securing taster weeks   *“I’m committing myself to another eight years of train-*  *ing and you don’t want to get that wrong”* | - The ability to take control of their time and also improving work-life balance, | - “Get off the treadmill” and look after own resilience and recharging. |  |
| Beattie 2017  UK / FY1 / Specialty choice – psychiatry  CASP 20 / Satisfactory |  |  | - Positive work-life balance in psychiatry, different with other specialties and more like a 9-5 working role, less rushed and more in-control.   *“Overall I have enjoyed it. It was really nice environment to work in, it’s really nice having like a 9–5 kind of job that’s kind of not too stressful and I have felt like I am in control of the situation. (Participant 3)”* | - Emotional stress of psychiatry jobs and whether they can work with it in the future.   *“When you’re with a patient and in particular a depressed patient…you feel emotionally drained at the end of it so I don’t know if I could cope with doing that all day for the rest of my life. (Participant 10)”* | - The placement facilitated more time to reflect and learn about the specialty, and the personality that fit different specialty, e.g. psychiatry need to be brave and make bold decisions   *“People who work in psychiatry they do need to be quite brave and make kind of bold decisions, and obviously like the patient who we saw in the crisis team setting sometimes having to go down the route of a Mental Health Act and these aren’t easy decisions.’ (Participant 13)”* |
| Sreedaran 2018  India / Psychiatry resident / Specialty choice – psychiatry  CASP 16 (recruitment, relationship, finding, valuable) / Satisfactory | - Clinical exposure and enjoying the rotation   *“P7: “I worked in the psychiatry department in my internship and I felt that was the most interesting part of my internship. I actually thought that I could continue working like this for the rest of my life””* |  | - Preferring lifestyle in psychiatry as less hectic like surgery |  |  |
| Woodward 2018  Sierra Leone / Junior doctor / Specialty choice  CASP 14 (method, design, relationship, analysis, valuable) / Satisfactory | - Exposure during clinical experience as a medical student and intern. Whether the job content fit themselves from personality perspective   “*I mean I’ve done rotations in all the disciplines…And I think [specialty] is like the most, it’s more me.” (JD2)”* |  | - Whether selected career allows close to their children etc. and some specialty training less time consuming thus allowing more time for family. |  |  |
| Cuesta-Briand 2020  Australia / PGY1-5 / Rural  CASP 17 (relationship, ethics) / Key |  |  | - Lifestyle factors, friendly people, less stress, a greater ability to maintain a good work-life mix   *“I don’t think I could live in a big city now. As well as you can get from one side of town to the other in ﬁve minutes when there’s no traﬃc. You’ve got everything that you need in terms of shops. I’m not much of a city person, so yeah, just the lifestyle, being outdoors, camping and things like that. (I09; Female; PG3)”* |  |  |
| Harris 2020  UK / FY2 / Specialty choice – GP  CASP 19 (relationship) / Key | - The reality of some work make them realize it’s not the right career for them; or realizing that some specialties are very challenging   *“‘I sort of thought that it would be something that would be well suited to me, in terms of what I like about medicine and I do like the kind of holistic care [. . .] Doing it now as a doctor has conﬁrmed it to me but I just don’t like it, it doesn’t . . . it doesn’t have everything for me if that makes sense.’ (FD21, CMT)”*  *“I actually think they have one of the hardest jobs, being a general practitioner. [. . .] But I’m very conscious of lots of people telling me that general practice isn’t what it used to be. [. . .] It sounds like a much higher-pressure specialty than it used to be.’ (FD23, undecided)”* | - Doing it and enjoying it, a powerful experience.   *“‘I am doing it now as a job and it’s great, I really, really like it.’ (FD8, GP)”* |  | - Pressure of GP life make the career less appealing. |  |
| Rizan 2019  UK / FY2 / Not going into specialty training  CASP 18 (relationship) / Key | - Not ready for decide which specialty to choose because of inadequate experience and exposure - Some felt that they do not have competitive enough portfolios, and taking F3 enable them to discover to reaffirm their interest. |  | - Taking time out to alleviate stress and exhaustion. Some were under pressure to work beyond 13 hours per day due to staff sickness, taking time out of training allowed individuals to regain their work-life balance with greater control over working hours. | - Taking time out to alleviate stress and exhaustion. Many felt that they were put in situations that they were out of their depth especially during on-call. They were unsupported by senior colleagues, did not have time to eat or use the bathroom. ‘it’s a bit like PTSD I suppose’ (2017;1), and ‘it was low grade and didn’t involve a psychiatric breakdown, but it was burnout by any other name’ (2015;1). Taking an F3 would help him to get his ‘mental health in a good place before carrying on’ (2017;1). - Reflecting their peers who didn’t take time out as “pretty miserable (2015;4)” - Participants felt a loss of control across a broad range of factors during foundation years, including physical location and rotas. Participants also expressed great diﬃculties in arranging study leave or annual leave, feeling ‘controlled by rota coordinators’ (2016;5). Being trapped on a conveyor belt of education from school, through medical school and onto postgraduate training, ‘training ladder fatigue’, ‘they just can’t face climbing another ladder’. Taking F3 enables to regain autonomy and do things of personal importance and own need.   *“a step oﬀ the treadmill I suppose, because that’s the thing, you can still work on your CV and you’re still a better applicant and you lose nothing ... ‘cause that year is a more enjoyable, fulﬁlling year for you as a human being.”* |  |
| Appleton 2017  UK / Psychiatry trainee / Specialty choice – psychiatry  CASP 20 / Key | - Having exposure to the specialty as med school experience very limited   *“P6 male CT1 “I think ifI hadn’t had those experiences during medical school… although I didn’t come away from this thinking, oh, I must definitely become a psychiatrist, it did make me think, well, I’d like to get more exposure to it, and it’s because ofthat, that I chose a Foundation programme with psychiatry. IfI hadn’t had the Foundation programme experience, I probably wouldn’t have decided to pursue it””* | - Feeling challenged to develop certain skills was appealing   *“P5 female ST7 “There was something quite skillful about seeing these psychiatric patients and actually establishing a rapport with them and getting them to the right team…. that was the different kind of challenge””* | - Healthy work-life balance having influenced their choice   *“P15 female CT3 “There seems to be a larger emphasis on work-life balance in psychiatry, they encourage people to take time out …do things that interest them… it feels like they actually value their trainees””* | - Feeling lonely during FY training in psychiatry training   *“P6 male CT1: “Being on call in psychiatry as an F2, you …feel a bit isolated from other doctors” “Most of the psychiatry training happens in separate NHS trusts…””*   - Emotional stress and the responsibility of making decisions | - Concern of losing medical skills and not being considered a “real doctor” or “proper doctor”   “P12 female ST6 “I think you get de-skilled quite quickly as a psychiatrist, because you don’t deal with physical health problems…You become more like a mental health specialist rather than a doctor... I think that discourages people from wanting to do it…they don’t get as much pride in being psychiatrists as…one would as a neurosurgeon.” |
| Essa 2010  SA / Doctors / General  CASP 17 (recruitment, relationship) / Key | - Exposure to different specialties and facilitated decision-making   *“I think it helps you decide what to do further in your career because you‟re rotating through almost everything – Participant T, F [HJ/RM]”*   - Elements of doubts and regret of choosing to continue with medical studies rather than something less challenging   *“Initially you tell yourself „why the hell did I get myself into this, I could do anything else, I should have done anything else, I studied six years for this, do I need this crap in my life' – Participant J, M [Bara]”* |  | - High workload and long working hours, though not directly linked with career decisions | - Emotional stress, depression and anxiety, though not directly linked with career decisions |  |
| Croghan 2020  Ireland / Intern / Specialty choice  CASP 16 (relationship, ethics, analysis, finding) / Satisfactory | - The “real-life” aspect and authentic exposure; expectation vs. reality   *“you try something and you like it, but then you have to ﬁnd out about it, what its really like to live it (Ollie)”*  *One recalled how ‘I initially wanted to do GP…[however] realised that I don’t like knowing bits of everything and much prefer to know one topic very well.’ One intern ’had previously decided on anaesthetics‘ but decided against it when ’working with intubated patients in theatre’ did not appeal, whilst another ‘enjoyed studying the theory of psychiatry’ however found ‘the placement emotionally taxing.’* |  | - Observed/reported lifestyles with particular specialties and future flexibilities |  |  |
| Firth 2011  UK / FY2 / Specialty choice – GP  CASP 16 (design, relationship, valuable) / Satisfactory | - Might have discounted GP in undergraduate but now changed   “‘I’m seriously considering general practice now and I wasn’t considering it at all before, so it’s been a radical turnaround for me, and for the other fella who was in – he was the other foundation in my practice, we both radically changed our ideas, he wanted to do hospital medicine and I wanted to do orthopaedics, and it’s, yeah, I mean it’s just completely transformed the views that I’d formed as a student.’ (Participant: Int 7)” |  |  |  |  |
| Liang 2019  Australia and NZ / Women leaving surgical training / others  CASP 19 (statement) / Satisfactory |  |  |  |  |  |

1. **Exploring, experiencing, and witnessing workplace and organizational norms through working with supervisors, senior colleagues, peers and the team, and the community**

|  | **Relationship with supervisors/consultants** | **Relationship with peers** | **Relationship with senior colleagues and the team** | **Relationship with patient and the community** | **Characteristics and hierarchy of career options and specialties** | **Workplace location, condition, resources and environment** | **Feeling valued by the organization and healthcare system** |
| --- | --- | --- | --- | --- | --- | --- | --- |
| Williams 2000  UK / PRHO / Specialty choice – surgery  CASP 17 (recruitment, ethics) / Key |  |  |  |  | - Certain characteristics of surgery like surgery as “doing” rather than “thinking”, taking a narrow view of patients rather than holistically   *“This is being a bit sort of generalising but I don't like the way surgeons think. I don't like not looking after patients, not getting to know them and that sort of thing. When you go on the ward round you just talk about the leg and whatever and there's someone sitting there I like surgery itself. I like the idea of it, but I just find that I get a bit irritated by surgeons, the way they think¼ most surgeons that I see, I see them and I think, I don't want to be like that. (PRHO 5)”*   - Gender as a barrier pursuing career in certain specialties like surgery as no female seniors   *“…but to go into a specialisation such as orthopaedics it's quite difficult, and I do fnd that a bit worrying…'Cos the registrars that have been through since I've been here, we've had two lots and they've all been men we haven't had any women. When I was at St J's (as a student) there were no female orthopaedic registrars there either. So yeah, it does concern me a bit because you might have all this hard effort trying to get to registrar level and then just get turned away because you're a woman, not a bloke. (PRHO 10)”* |  |  |
| Scanlan 2018  UK / FY2 / General  CASP 20 / Key | - Attracted to specialties where there are enthusiastic and supportive supervisors who are willing to take an interest in the professional careers of FP2 doctors and helps to make it an enjoyable experience, feeling valued, committed to look after their professional development and wellbeing.   *“I think senior support at times was a huge factor for us, particularly in A&e. I'm never on shift without somebody who’s at least a senior registrar or a consultant. …When I went to A&E, my educational supervisor in A&E, she’s very proactive. she’s super. We did a lot of discussion around how I manage patients, so she’ll call the patients up that I’ve seen X number of weeks ago, and we go through and look at my documentation, see how I managed it. And it’s not criticising me. I mean if it is critical, it’s constructive criticism. so I think that’s a huge, huge aspect of*  *learning as a junior. And that’s the first time I’ve had a supervisor that has been that enthusiastic, and that makes a hell of a difference … She even creates*  *opportunities for you…. And then that leads into feeling valued. (Story 1 Daniel)”* |  | - Feeling a valued member of the team, the seniors are eager for interns to learn on the job.   *“… when I got out I was actually doing the job because I could have easily just been given rubbish tasks to do for 4 months but I was encouraged to take part and work with a wide variety of consultants, see how they worked and becoming, like, familiar. (Story 2 Steven)”*   - Other seniors or team member are not supportive and blame interns for their faults.   *“And there’s huge neglect of training for foundation doctors, especially at FY1 level. I don’t think that you’re respected or considered to be a practising clinician. I think there’s not much in the way of recognition for the work that you do. And I think that you do a lot of legwork for a lot of other people. And people aren’t very thankful of that. I don’t think that you’re treated well by other members of healthcare professionals within the teams. And that makes your role feel devalued as well. You feel devalued in that sense that other people don’t treat you very nicely at all. (Story 3 Abigail)”*  *“I did have a case, when I was in paediatrics, where I didn’t feel supported at all by my seniors. In fact, the opposite, by the consultants, and it really upset me and got me down and affected me for months, so… And at the end of that, I thought, no, I don’t want to. I decided to take a year out from that. There was a patient that became seriously unwell, that I didn’t know that the baby was unwell, and then got, kind of, blamed for it afterwards, despite the fact that I tried to escalate it and they didn’t really, like, want to take any kind of responsibility. And I tried to get some help and no one would come to help. And then I, kind of, got blamed for it and not supported through it, not debriefed or… I just, kind of, felt like it was my fault and then I had to kind of process it and deal with it on my own, rather than having… We have our clinical and educational supervisors that are meant to help us through those, like, the emotional aspects of our work, as well as all the practical aspects of just getting through the course. But it, it all, they’re all consultants that take on that role, so… And obviously, every personality differs, and everyone… so I was just unlucky that all of the consultants there didn’t really seem to care about that. And there wasn’t anyone that I felt like I could ask. (Story 4 Gemma)”*  *“I would have said, there were certain bits that have made me think, I’m 100% positive I don’t want to continue working in this hospital. And I would have said… My second job in the FY1 was {a surgical specialty}… There was meant to be two FY1s and two FY2s. The FY2s were on the registrar rota, which was hell on earth for them, and therefore I was the only FY1, and there were no FY2s because they were on the reg rota. And I was put in horrific, horrific situations… there was one horrendous weekend where I had three people in the HDU, had sixty patients, and then I had another four beds due where I had four people that were really sick with pneumonia. And I hadn’t had a medical job, and I didn’t know what I was doing, and I have never in my life felt so overwhelmed, and I just… I hated it, I absolutely hated it. I didn’t get support; I’d phone the registrar to come in, they came in in the morning for an hour, did a ward round and just left. And I was left on my own for a full weekend”* |  |  |  | - FY doctors feeling disheartened by the NHS and never expected to be treated so badly as a professional. The NHS does not value or care about their wellbeing. Consider FYs as a hassle or a problem.   *“I don’t think if you went to any other institution, any other organisation, anywhere as a professional, or in any other business they would make you feel crap in the first… You know, having that first experience, you know, I mean, you know what I mean to work somewhere in the first year. You just feel totally disengaged, and very down trodden. And you feel undervalued. I found it really disappointing. Like, I’ve spent years training to do this job. And I really, really wanted to do it. I’ve always wanted to be a doctor. And I got here, and I’ve just found it devastating. I’ve really been so disheartened, and just put off with the profession as a whole just because the way in which the work is.’ (Story 3 Abigail)”*  *“the thought of actually having to spend any more time in that hospital is just horrifying, but I think…So the thought of having to do six, seven years to consultant, there’s no way. there’s literally no way I would do it. Management don’t know who I am, don’t know what I’m about. And if I raise a concern, I think they see that you’re a hassle, it’s a problem, as opposed to, you’re a valued team member that they think is worth being there. I don’t, yes, I don’t feel valued in that capacity. (Story 5 Clare)”* |
| Alberti 2017  UK / GPSpR / Specialty choice – GP  CASP 16 (recruitment, relationship, analysis) / Satisfactory | - Role models influencing perception of GP   *“So I think role models is what changes perception, we need people to stand up and help change things"* |  | - Hearing consultants, junior doctors and nurses criticising GPs for example for “rubbish” referral   *“I think one of the reasons why I didn’t just apply for GP straight out was because the people, the medics that I was with were saying, well you’d be wasted you should be doing medicine … and they tipped me away from where I’ve actually ended up, if that makes sense”* |  | - GP being considered as very simple, not using or possessing particular skills, failing to independently manage medical problems, frequently referring   *“GP’s just being very simple, managing very simple things and you’re not going to be using your brain that much, you’re not going to be using your clinical skills that much it’s just talking and talking. (Senior registrar)”*   - “Just a GP” frequently reported when trainees are discussing career options with more senior clinicians. They would even use this term themselves – linking with the idea that GP seen as inferior to hospital specialties.   *“you’re too good for GP’ - like that was kind of what he was getting at.”* |  |  |
| Merrett 2017  UK / FY1 / Specialty choice – GP  CASP 16 (design, relationship, analysis) / Satisfactory | - Witnessing characters of poor role models during placement within primary care, do not want to become such doctor (unclear if this was in medical school only or also refer to internship) |  | - Witnessing GPS being stressful and burning out   *“Yeah. I’ve come across GPs that are going through, like, litigation processes, and have been absolutely burnt out with it. I think everyone at some point, whatever specialty, will miss a diagnosis. But you’re much more open to litigation in general practice.’ (Participant, group 3)”* | - Ability to provide continuity of care spanning many generations in GPs, building doctor-patient relationship   *“‘I like the fact that I could build up a picture of the family dynamic. Then you’d always have something to talk about from their last appointment, and I think it’s more enjoyable when you can see the work you’ve done is going well, and the fact that you can remember them makes the next consultation easier, because you’ve already got a foot in.’ (Participant, group 4)”* | - Lack of respect for GPs. Interns ashamed to mention it in the hospital environment for fear of prejudicial treatment. Negative comment of GP “everywhere”   *“I’m on general surgery, and I think surgeons, they do not respect GPs. I think they kind of undermine the GP’s potential or achievement in life. They say: “Oh, you only do GP if you’re not smart enough.” I remember when I was on my ward round, a surgeon just said to me: “The people who do GP are the ones are like the bottom of the year”, and I just looked at him. I was so shocked when he said that, because that is just an inappropriate comment.’ (Participant, group 5)”* |  |  |
| Smith 2018  UK / FY1 / Migration  CASP 20 / Key | - Having consultants as mentors for providing career advices   *“My consultant [in a previous job] was very good in encouraging and looking at options and she was probably the person who gave me the most advice about careers. I found that immensely useful, so I think to have some kind of continuity with someone who could be a mentor would have been probably very helpful. (P8)”*   - Bullying from consultants make F2s want to leave the UK as the feedback they get is Australia and New Zealand is different   *“I was sitting flicking through a massive set of notes and then the consultant walks round the corner and goes; ‘What are you doing?’ and shouts at me in front of all the nurses, everyone on the ward. Then when you get a bit tearful tells you to grow up in front of everyone, very publically. Numerous occasions like that, particularly in surgical jobs. Just being made to feel that you’re never good enough and never getting any thanks for what you do. I don’t know for sure that that’s different in Australia, but from all the feedback I’ve got from everyone that’s in Australia and New Zealand working at the minute it appears to be quite a bit different. (P2)”* | - Other junior doctors being viewed as important social relationship. Lack of peer support led to loneliness and poor enjoyment.   *“‘You're working with the same big bunch of people. So, you get to know quite a lot of people. It was a really good sized job actually for me, for making friends and creating like a social circle. I actually did quite enjoy it.’ (P13)”*  *“I think also it’s been quite a lonely job because you are by yourself a lot of the time. We don’t have F1’s. We don’t have registrars. I didn’t realise how much that can affect your job satisfaction… …because thinking back all the other jobs I’ve had have been in really big teams and perhaps even when we were busy and it was maybe a bit stressful you could moan about it to somebody else and the loneliness affects you I think…(P1)”*   - Leaving the UK will support their peers as there will be less competition for them   *“If you look at people leaving as a whole it will make the jobs easier to get in some ways because they’ll have less competition. It's different if you're leaving a rota, if you're already in a training position, but it shouldn’t affect my colleagues because I've no obligation to be there in that hospital. (P7)”* | - Senior colleagues like registrars not being supportive   *“In neurosurgery the registrars weren't very… they’re not a very supportive bunch. There’s someone suddenly blowing a pupil and you're like, is this person dying? What am I going to do? They’d be like, ‘I'm busy, sort it.’ (P3)”*   - Poor relationship with the nursing staff affected the enjoyment at work   *“The nursing staff wouldn't listen to me. They would then go and get consultants and run everything I did past them. There are some strong characters in that department and it’s well known that that is the case. I just clashed with them and I find it quite condescending and made me feel like, that you weren’t a doctor, that you are more a medical student because everything you said had to be verified by a consultant. (P9)”*   - Unfavourable experiences working with locum   “Sometimes you can get people that are completely new to the specialty and healthcare around the UK or even healthcare at all and that can be more challenging. They’re at my level and they’re filling up a gap in my rota slot at my level but at the same time I find them depending on me…which can be a bit difficult. (P16)” |  |  | - The working environment and morale within the NHS being considered as poor, whereas the working environment in Australia and New Zealand is considered more pleasant.   *“There's just a lot of kind of negativity in the NHS at the moment amongst junior doctors and I don’t know if it's become a bit of a culture thing as well… We have great resources, a great structure, but something at the moment isn't working. We don’t have enough money or we don’t have enough doctors and it's just a horrible environment to work in I think. (P8)”* | - F2 doctors think Australian and New Zealand healthcare organizations value their staff more than the NHS. Lack of support from NHS to help resolve work-life conflicts,   *“How often have you stayed overtime and got no…no one thanks you for it. You get upset because you’re hungry, tired, haven’t gone to the toilet and you’re never getting paid for it. (P10)”*  *“I suppose medics are viewed in a better way [in Australia], just more respect. The patients probably pay a bit of insurance and they do have to take accountability for their own health care and that way they do then value all the healthcare professionals more, not just the medics.’ (P6)”*  *“We know for a fact that we have three vacancies coming up in our rota, and they’ve done nothing to try and fill them, because in the end they're just going to email round us, rather than getting a locum… and the thing is we do, we do still fill it because we need to, the patients need us.’(P9)”*   - F2s unsure about how to raise concern and they might be penalized for doing so   *“How are you supposed to give any of this feedback to the top people, because where on earth are they? I think also sometimes you're worried if you had a concern and you expressed it, that number one, it would fall on deaf ears, and also would you be getting into trouble for expressing concerns? (P9)”* |
| Spooner 2017  UK / FY2 / Specialty choice  CASP 18 (relationship) / Key | - Consultants being mentors engage and inspire interns, being supportive and help interns rebuild their damaged confidence   “One of the consultants… went through my portfolio with me… he’d bring me along, he’d teach me, he’d let me get involved. He was absolutely a mentor. (GP0P17)”  “In my GP placement, had an amazing supervisor that was just really supportive and gave me feedback how it should have been given, and just kind of coaxed me through and built up my confidence again. (GP0P2)”   - Or in sometimes, supervisors inadequately supported interns for difficult work.   *“In stroke, the consultants were there in the morning, for an hour, for ward rounds, and then disappeared… whereas in a lot of other specialities, they gave us their mobile numbers, or they said, don’t hesitate to contact us… similarly, our SHOs… took a bit of a step back. And I just didn’t feel as supported… It was very hard work, it was very intense. (GP0P17)”*   - Consultant being supportive of GP career choices and also psychiatry choices. |  | - Getting familiar with training requirement, working practices, and opportunities with different specialty careers while working with specialty trainee, which is important in informing specialty decisions.   *“The taster I think helped me because it gave me an idea of what… the kind of people would be like, see what the patients would be like. That, kind of, made me think—yes, I could probably do this… I really felt like they tried to, like, integrate me into the team.’ (GP0P11)”*   - Interns feel drawn towards supportive teams who engage and inspire them or help them rebuild their damaged confidence   *“I met a few people there [psychiatry] who were really encouraging and also were really passionate about the work… I’d say that was the one thing where there [were] people involved that I thought this is what I want to do. (GP0P11)”*  *“I’d like to be around a caring, friendly, supportive team, because that will… I feel like that will make me more caring, supportive and friendly, whereas, in a more direct blunt specialty, that’s…I’ll definitely become more like that and it’s not someone I want to be. (GP2P3)”*   - Working in supportive teams could transform a new or worrying situation into positive experience. - Witnessing registrars that are “broken” and consultants present until 23:00, interns switch career plan to a different specialty to avoid extended commitment. - Appreciate working in teams where colleagues demonstrate similar preference and reject teams who act differently   *“They weren’t interested really, it wasn’t their job and I don’t really want to be like that, I’d rather listen to what the patient wants and adapt than just stick on my road. (GP2P3)”*  *“Consultants… junior doctors that I’ve worked with, the registrars and the SHOs, I’ve felt like they’re quite similar to me… they all cared a lot about the patients, they saw them as people not just disease processes and listened. (GP1P6)”* |  | - Some specialty training programme being viewed as highly structured, intensive, competitive and demanding whereas other being rated as unworthy or unexciting   *“A lot of the best candidates go for very competitive specialties… because a lot of medics are competitive, by nature they’re very driven people and they like to do the best thing; so when something is portrayed as a lesser thing then I think almost psychologically they’re less inclined to go for it. (GP1P4)”*   - GP being viewed as nothing better than a reserve option by both interns and specialists. Interns had kept quiet to avoid being badly thought of or excluded from specialist training.   “*There was an image of a GP as being somebody who’s, kind of, failed every other speciality, not able to get into a speciality, so they’ve given up, they’ve become a GP… and it subconsciously roots into your mind. (GP1P13)”*  *“When I told her [oncologist] I was going to be a GP, she looked at me and she said, oh, are you pregnant? (GP1P16)”*  *“Oh, why do you want to do [GP]?’… it just seemed a boring pursuit for them… it put me off a bit. I mean because I didn’t want to be thought of as the one who wasn’t trying hard… or wasn’t going to like put their hand up for something that maybe wouldn’t be relevant to my future. (GP1P14)”* |  |  |
| Ludwig 2018  Germany / Practice year / Specialty choice – GP  CASP 16 (statement, relationship, ethics, valuable) / Satisfactory |  |  |  | - Working with the community and able to being integrated into a rural community   *“Well, I think, with a job that [...] really brings personal benefits for the locals [...] I think it’s easier. (TN 5, Post, WB-, Rural-)”* |  |  |  |
| Parker 2014  NZ / Final year undergrad / Specialty choice – GP  CASP 20 / Key | - Contacts with general practitioners being the biggest influence for attitude towards GP. Observing GPs with good practise and excellent bedside manner vs. observing poor practice (less well regulated). |  | - Other junior doctors who want to pursue GP as a career and GP trainees as potential role models. Their behaviours also influence interns’ decision.   *“There are the house surgeons that want to be GPs… they make you realise that it’s not just lazy ones that want to do general practice. They are actually a lot of talented, smart, good doctors. (#6)”* |  |  |  |  |
| Olsson 2019  Sweden / Young doctor / Specialty choice  CASP 17 (recruitment, relationship) / Key | - Encouragement and lobbying from supervisors can impact interns’ choice of specialty   *“‘Well, probably, yes, but there were many mentors that you had when you went ’round to different departments who were really like, ‘Seriously, you should start in our specialty’! There was a lot of lobbying, like in paediatrics or gynaecology; ‘We need more men here. You should apply’! or ‘You’re good with children. You should start here in Paediatrics’. That kind of thing. Certain specialties are really persuasive’. (No. 3, internal medicine, man)”* | - Interns socially both professionally and privately with other doctors, and the former include also meetings and other events organized by specialty associations, the union or network set up for future specialty training. - Competition between peers on certain specialty, one must elbow one’s way into the theatre to become a good surgeon. This sometimes put interns off.   *“‘At the surgical and orthopaedic departments where I’ve been, you need to be fairly assertive, even bullish, to somehow get the educational experience you need. You have to make sure you get into theatre, struggle, really, and hinder others in your way to becoming a specialist. And that was not something I had any desire to do. Being somewhere where there was a lot of competition, I wasn’t interested in that. At all’. (No. 5, internal medicine, woman)”* |  |  |  |  |  |
| Querido 2018  Netherlands / Final transition year / General  CASP 20 / Key | - Specialty clinicians being role model or mentor and “want to be like her”   *“One of the general practitioners during my internship, she was a role model for me. How she treated and communicated with her patients, her knowledge, everything she did. I really saw an example in her and I want to be like her when I become a general practitioner myself. (no.14, male)”* | - Student or peer-initiated information collection, extra-curricular activities medical students proactively undertake to gather information to make a career choice, such as voluntarily shadowing doctors in a preferred specialty, visiting medical career events or workshops, participate in a medical career event committees. These information provide more information or the possibility to check their personal questions with someone with more experience or knowledge. | - The characteristics of team and colleagues are important. Interns sought to be part of the team, to participate in the teamwork, to have colleagues and to identify with the features of colleagues.   *“…Yes, this specialty really got me, I liked that. I am attracted by a nice team and this was a nice team.” [..] Yes, the entire team, the gynaecologists and the residents together. I did not experience that before, where people respectfully work together and at the same time enjoy work and are not too serious all day. I feel at home with them. […] I do like doing things on my own, but I really want to work in a team, definitely. (no.6, female)”*  *“…I think it is also the colleagues that I see, they are like me. So, it is the combination of the specialty and the colleagues” (no.20, female)”* | - Characteristics of patients and previous experience working with patient contacts   “*…I just like old people. As a student, I worked a year in elderly care and that really brought me pleasure. I enjoy being in contact with those old people. (no.2, female)”* |  |  |  |
| Brodribb 2016  Australia / Junior doctor / Rural practice  CASP 18 (relationship) / Satisfactory | - Positive experience and support from supervisor strengthened positive attitude towards rural practice. Lack of support negatively impact the likelihood of rural career choice.   *“I certainly didn’t [feel out ofmy depth]...not in [regional town] because it’s a bigger centre so in the general practice you always basically talked about whatever you’d seen with your supervisor. In the emergency department...we were always supervised and supported by the staff there. (F, 26, Registrar, Z)”*  *“The surgical one [intern] was challenging, but the emergency rotation [PGY2] was a very bad one. It was very unsupported. As someone who had been a doctor for slightly over a year, I was the most senior person in the emergency department overnight and asked to supervise two people who were more junior than myself... (F, 27, PGY2, Y)”* |  |  | - Living experience in regional city changed interns’ perception of where they want to work. Enjoy the friendly people.   “*The people were more friendly. The hospital was more friendly than the city hospitals...Rather than feeling like a stupid medical student like I did in the city, I felt like part of the team...and then I stayed on for another year and actually my husband and I bought some property nearby, so we’re still part of the time in the city and part of the time out there. (F, 34, Registrar, Y)”* |  |  |  |
| Van Hamel 2020  UK / FY2 / Not going into specialty training  CASP 20 / Key |  | - Comparing with peers who take time out of training, some interns may feel they are disadvantaged if they did not do that, they would be missing out positive experiences, or earning more through locums – a “cultural norm” - Positive recommendations from those who have experienced an FY3 themselves. |  |  |  | - Poor rest facilities, limited access to catering facilities and expensive, limited parking impacted on their feelings of being unappreciated. This negatively impacted on doctor’s feelings of self-worth. Doctors felt that the pressure put on them in the workplace from workload, pressure to cover rota gaps and poor facilities increased their frustration at work and negatively impacted on their feelings of being appreciated | - Feeling unvalued that they were only valued for service provision. “Cheapest option” to fill rota holes,   “you are doing two people’s job and also kind of undervalued because of that”   - Frustrated by administration leading to losing motivations. “you know nothing is going to be straight forward, you are going to have to chase people around”. Even those who felt motivated to request study leave or try to work towards outside goals were frustrated by the administration processes required for these and sometimes unable to achieve these goals because of this. - Lack of support from the foundation programmes with regard to options available, retruning to work etc. |
| Beattie 2017  UK / FY1 / Specialty choice – psychiatry  CASP 20 / Satisfactory |  |  |  |  |  |  |  |
| Sreedaran 2018  India / Psychiatry resident / Specialty choice – psychiatry  CASP 16 (recruitment, relationship, finding, valuable) / Satisfactory | - Psychiatry role model |  | - Influence from seniors and discussing/seeking information from them   *“P11: “I had considered it as an option but not the first option. But later on, when I spoke to my seniors and some of my family friends who had been in psychiatry, had taken up Psychiatry already I realized I can do it. So then I chose psychiatry because of that””* |  |  | - The process of postgraduate medical selection and preference for central institution training |  |
| Woodward 2018  Sierra Leone / Junior doctor / Specialty choice  CASP 14 (method, design, relationship, analysis, valuable) / Satisfactory | - Role models are important sources of inspiration and gaining a preference for certain clinical specialty. Nothing what they say but also how they behave.   *“What motivated me to [clinical specialty] is that I mean the [specialist] that we have now, Dr [x] he’s very good. When you’re posted to Dr [last name] the [specialty] he would explain. It was so simple, it was so easy, you would understand everything. (JD12)”*  *“Almost all [specialists x] in our hospitals here and at COMAHS, they are very nice people, unlike the [specialists y] people. They have some attitude problem. Some of them are like proud. (JD4)”*  *“I’m not the [specialist] type of person so I’m not going into [specialty] (JD8) ”*  *“It’s [specialist] more of who I am; It fits into the kind of doctor I think I am (JD2).”* | - What other peers choose to do and realize a gap in the country   *“Because so many people [junior doctors] want to do [clinical specialty X]. And there’s nobody who wants to do [clinical speciality Y]. And you see your country growing backward. Someone has to take that risk or let me say to specialise in [clinical specialty Y]. (JD11, Oct 14)”* |  | - Witnessing the need of the people and decided to serve the community   *“If there’s anybody who needs healthcare it’s mothers should come first. Because they are like providing the world for us. So I saw [in my internship] the types of pains they [mothers] go through. And I had a change of mind that no I should stay here to help. (JD14)”* | - Different popularity of specialties and the need to fill the gap for less popular ones   *“Because so many people [junior doctors] want to do [clinical specialty X]. And there’s nobody who wants to do [clinical speciality Y]. And you see your country growing backward. Someone has to take that risk or let me say to specialise in [clinical specialty Y]. (JD11, Oct 14)”* |  |  |
| Cuesta-Briand 2020  Australia / PGY1-5 / Rural  CASP 17 (relationship, ethics) / Key |  | - Positive interpersonal interactions with peers and senior staff, being familiar with people around them and developing a sense of community. Wanting to maintain already established personal relationships. - Such relationship extend beyond workplace, some personal friendship and socializing.   *“I was very familiar with the place. I actually knew a few people working there already. It seemed like a nice place to work and I quite enjoyed being at [urban hospital], so I thought I might as well just stick around for a bit longer while working as well. (I16; Male; PGY3”*   - Peers as valued source of support and influence career decision-making as they are the main source of information on training and work opportunities. | - Highly value supportive and friendly teams, approachable senior staff, easy access to consultants. Smaller teams with better personalized training.   *“I opted to go to [rural location] under the surgical team for my surgical term and really, really enjoyed the smaller hospital, the more intimate and close-knit working environment and the smaller teams and how they kind of worked and do well together at ﬁrst-name basis. When I went back to Perth to this big tertiary hospital with a whole lot of people who don’t know your name, it’s not as personable so to speak. I really missed and longed for that small work group that I experienced in [rural location]. (I06; Male; PG2)”*   - Career information and advice received usually from colleagues, personal network as there is an information gap, the information was “out there” but not easy to find. - *Career advisors and mentors* are providing a valuable link to choosing career (though unclear who are those people) |  |  | - Supportive work practices, and ﬂexibility of contractual arrangements positively inﬂuenced doctors’ experience of their workplace | - Perceived administrative assistance inﬂuenced doctors’ experience of their workplace |
| Harris 2020  UK / FY2 / Specialty choice – GP  CASP 19 (relationship) / Key |  |  | - Some seniors being very supportive of junior’s career plans   *“‘No senior would ever say, “Don’t do that. That’s a terrible specialty.” If you show interest in anything, they’ll support that regardless of what that specialty is.’ (FD2, surgery)”* |  | - Criticism of GP from general practices.   *“A lot of the surgical registrars were dismissive of GP referrals and what they thought of GPs in general was very negative. (FD9, GP or psychiatry)”*   - However banters of specialties is seen as a natural consequence of passion for one’s own specialty, not something that affect their own opinions   *“It wouldn’t matter to me about a reputation or what people think of a certain specialty. If something interested me, it wouldn’t bother me what other people thought about it, I’d just do it.’ (FD17, undecided)”*  *“‘I think there are certain specialties that get made fun of. It hasn’t really had any impact on my decision making. I think probably because they get made fun of, partly, because people are jealous.’ (FD24, anaesthetics)”* |  |  |
| Rizan 2019  UK / FY2 / Not going into specialty training  CASP 18 (relationship) / Key | - Being warned against the concept of taking time out of training by consultants, being told “it would be career suicide (2015;4)”. However for others, their consultant were supportive. | - Career choices validated by their peers who are similarly taking the same one, finding this reassuring. Whereas participants who started earlier when it was not yet a norm were less certain and nervous.   *“I would have been an odd one out, if I’d gone [straight] into training (2016;1)”* | - Not supported by senior colleagues also due to staff shortage. |  |  | - General morale appeared low at workplace because of the stress and exhaustion. | - Junior doctors feel undervalued and under-appreciated. Participants described being treated ‘like a ward mule’ (2015;2) or feeling used for ‘service provision’ (2017;2). |
| Appleton 2017  UK / Psychiatry trainee / Specialty choice – psychiatry  CASP 20 / Key | - Consultants not being supportive of career choices/bashing psychiatry |  |  | - Enjoying the opportunity to work with patients and involvement of the psychosocial aspect of their care | - Negative experiences and some stigma towards working with people with mental health difficulties   “*P17 female CT2: “There is always a stigma. In my medicine F2 post ... I had just finished psychiatry and when I said that I wanted to do psychiatry my consultant said something like ‘then there is no point bothering with you then’.”…”* |  |  |
| Essa 2010  SA / Doctors / General  CASP 17 (recruitment, relationship) / Key | - Poor supervision leading to emotional stress and limited opportunity to learn, though not directly linked with career decisions |  |  |  |  | - Wil be willing to work in the public sector if better salary, improved working environment and less bureaucracy | - Sufficient amount of funds being allocated to the public healthcare sector but that these budgets were being seriously misused. Hospitals are not being adequately upgraded; much-needed equipment is not being repaired and essential resources are not being purchased or adequately distributed. There was also a sense of despondency from participants as they mentioned that there was a lack of accountability from people in senior positions, thus it became a frustrating environment to work in and provided reason for healthcare professionals to leave public hospitals   *“Government giving us budgets…people misusing the budget completely. We’re in the stock-room - look at this equipment, it’s from the 1980s! From the 1970s, „60s! I mean look at that machine – that’s probably older than both you and I, our ages put together! But its just you don’t get the support from your seniors…there’s no stock. You want a drug and there’s no stock, you want a drip and there’s no needles, its very frustrating in that kind of sense that your superintendents and your higher up who are running the place…ja so that’s why everybody is leaving – Participant D, M [Bara]”* |
| Croghan 2020  Ireland / Intern / Specialty choice  CASP 16 (relationship, ethics, analysis, finding) / Satisfactory | - Consultants as role model and highly influential in career choices - Open encouragement, observed skills/satisfaction, personal interactions with consultants and senior doctors; or on the contrary, discouraging (due to lifestyle factors, poor training schemes), negative personal interaction, bashing other specialties, consultants themselves being dissatisfied, or having poor lifestyle |  | - Rapport with the clinical team, good relationship led to good experience and increase opportunities   *’Actually the three main, very different, specialties I’ve been interested in…the common factor was really good experience on rotation and good fun with the team’ (James). Good rapport was associated with increased opportunities, such as being ’brought along to theatre’ (Sarah). The structure of clinical placements affected students’ abilities to foster relationships, ‘if you’re only on a specialty for one week you don’t really get to know the team’ (James).*  *“the environment and doctors in some specialties are so negative that I would be completely opposed to…the specialty just based on the hostile and negative people that populate it.”* |  | - Bashing particular specialities, “one had been told her chosen subspecialty was ’not worthwhile or proper surgery,’ whilst others perceived that ’GPs are considered lesser doctors’ and that ’everyone moans about [surgeons].” |  |  |
| Firth 2011  UK / FY2 / Specialty choice – GP  CASP 16 (design, relationship, valuable) / Satisfactory |  |  |  |  | - Hearing bashing from hospital doctors and senior colleagues, their own mind being reinforced by the interaction   “*When you’re in conversations with medics or surgeons they would be saying – every now and then, they would say GPs do this but that’s wrong, this is the way it should be done, or this is such a rubbish referral from a GP or why on earth did the GP send this in and then you start to get the same sort of opinion.’ (Participant: FG10)”* |  |  |
| Liang 2019  Australia and NZ / Women leaving surgical training / others  CASP 19 (statement) / Satisfactory | - Insufficient role model leading to leave surgical training   *“...once I left [my sole female role model] where I did my intern years... I actually didn’t have another female consultant until I was a registrar [specialty trainee]. So all through my PHO [non-training registrar] time, they were all male consultants so it wasn’t even an option. (Participant B)”* |  |  |  | - How gender aspect of lifestyle, sexism, discrimination, harassment lead to women leaving surgical training, though unsure if they are during internship training or later |  |  |

1. **Worrying about future – will I get a job and get advanced**

|  | **Job market polices and changes, job security: will I get a job** | **Future training and professional development opportunities: will I get advanced** |
| --- | --- | --- |
| Williams 2000  UK / PRHO / Specialty choice – surgery  CASP 17 (recruitment, ethics) / Key |  |  |
| Scanlan 2018  UK / FY2 / General  CASP 20 / Key |  | - Choosing specialty because of the perception of support that would be available in the future, level of commitment from seniors, evidence of the level of investment and value the organization places on personal and professional development   *“I’ve done it and I liked it… When you see what they do and how they… how well-supported they are as a junior. So they’re probably the best trained in terms of consultants with juniors. so you’re one-on-one, junior and consultant most of the time as an anaesthetic trainee. The training you get is… You know, they’re able to deal with nearly everything, in terms of managing patients. That’s what attracted me to it as well is the quality of training you’re going to get. (Story 1 Daniel)”* |
| Alberti 2017  UK / GPSpR / Specialty choice – GP  CASP 16 (recruitment, relationship, analysis) / Satisfactory | - Negative comment about choosing GP due to uncertain future of the NHS   *“anyway my consultant was trying to discourage me from getting onto the GP programme, saying that, it might be appealing now but he doesn’t think that things will remain as such in the future”* |  |
| Merrett 2017  UK / FY1 / Specialty choice – GP  CASP 16 (design, relationship, analysis) / Satisfactory | - Uncertainty of workload and the impact of government cost-cutting on GP salaries, handing over clinical work to other healthcare staff, uncertainty about increase in privatisation of the NHS   *“It’s the whole: “Are GPs going to work weekends? Are they going to work evenings? Is it going to be 24 hours a day, 7 days a week?” With most of the hospital specialists you expect that to an extent, but while it’s all in flux I think it’s a bit unrealistic to expect lots of people to want to go and do it when you don’t know what you’re getting, letting yourself in for.’ (Participant, group 3)”* | - Uncertainty about the length of GP training |
| Smith 2018  UK / FY1 / Migration  CASP 20 / Key | - Proposed changes to junior doctor contracts in England provoke mixed emotions   *“I always wanted to work in London, so if I was applying for CMT1, I would have liked to have apply down South. [But] there was a lot of uncertainty and there’s still a lot of uncertainty now about what the contracts are going to be like. I think they have started releasing the provisional contracts now and they look terrible. You’ve been paid less and you’re working a lot more and you’re working one in two weekends. It didn’t seem like a good thing to trade rather than working somewhere where there is a good work life balance and sunshine and people appreciate what you do for a living in Australia—why would I want to move to England? (P12)”* | - Perception of better formal teaching in Australia than is provided in the UK   *“One of my friends who is working out in Perth [Australia], has said actually she has like hourly sessions kind of every other week where she’ll get bedside teaching. That's way above and beyond what I've had. I don't think I've had bedside teaching beyond what happens on a ward round. (P13)”*   - Concern of getting jobs that no one else want, low-quality job   *“My impression is that basically you end up with the jobs that no-one else wants from Australia or New Zealand. Quality-wise you’re probably ending up in a job that is less good than you would get in the UK but as a friend of mine put it to me you’re not going there really for the clinical exposure… most people are going there because they want a different experience.’ (P1)”* |
| Spooner 2017  UK / FY2 / Specialty choice  CASP 18 (relationship) / Key | - Changes to junior doctors’ contract increased career uncertainty leading to shifts in ST programmes’ attractiveness, e.g. more interest in GPST as they have shorter training programme and also more broadly as a profession   *“Three years’ training is the minimum which they [GPSTs] could do to get a job which would then allow them that freedom to either move or to determine their own contract… So I think if anything perhaps the contract has pushed people towards GP just because of the training period, giving them perhaps freedom a little bit earlier. (GP2P12)”*  *“I’m just a bit worried that the NHS is such an unknown at the moment in the future and… that’s my whole career… But I feel more and more that these people who work in the government are not really respecting us as a profession. (GP1P8)”*  *“With the way the current contract changes and the way the current health service is, I don’t think I’d want to work in an acute specialty anymore because I just think that’s the way to a burnout. (GP1P1)”* |  |
| Ludwig 2018  Germany / Practice year / Specialty choice – GP  CASP 16 (statement, relationship, ethics, valuable) / Satisfactory |  |  |
| Parker 2014  NZ / Final year undergrad / Specialty choice – GP  CASP 20 / Key |  | - Shorter training schemes sounds more attractive |
| Olsson 2019  Sweden / Young doctor / Specialty choice  CASP 17 (recruitment, relationship) / Key |  |  |
| Querido 2018  Netherlands / Final transition year / General  CASP 20 / Key |  | - Whether there are chances to obtain a residency position as some specialties are more competitive and popular, or have higher entrance criteria. |
| Brodribb 2016  Australia / Junior doctor / Rural practice  CASP 18 (relationship) / Satisfactory |  |  |
| Van Hamel 2020  UK / FY2 / Not going into specialty training  CASP 20 / Key | - Worry about becoming unemployed or having no confirmed role within a year, reservation on impact on their pension and future pay. | - Feeling instead of being trained for a future career, more towards being responsible for mundane jobs and leaving little benefit for personal development   *“especially in my FY1 a lot ofthe tasks I did almost felt administrative and I wasn’t learning or using what I’d learnt throughout medical school.”* |
| Beattie 2017  UK / FY1 / Specialty choice – psychiatry  CASP 20 / Satisfactory |  |  |
| Sreedaran 2018  India / Psychiatry resident / Specialty choice – psychiatry  CASP 16 (recruitment, relationship, finding, valuable) / Satisfactory |  |  |
| Woodward 2018  Sierra Leone / Junior doctor / Specialty choice  CASP 14 (method, design, relationship, analysis, valuable) / Satisfactory | - Choosing public health degrees to become more employable. |  |
| Cuesta-Briand 2020  Australia / PGY1-5 / Rural  CASP 17 (relationship, ethics) / Key | - Contractual arrangement and sustainability of certain specialities also influenced career choices although to a less extent - Choosing specialties that allow interns to “live in the country”   “*To a certain extent, my choices—sort of anaesthetic, general surgery, GP surgery type of thing—are very much based on the idea that if I went and tried to do ENT surgery, I’d never really be able to be in the country. And I’m not a big fan of GP work because I don’t like to sit down for most of my day. But part of what I’m trying to do is ﬁnd a specialty that would allow me to live in the country. (I04; Male; PGY3)”*   - Uncertainty about the future of rural GP anaesthetic model, whether this could hinder their future hospital work   *“I’ve deliberately ruled out doing GP anaesthetics or GP obstetrics because I’m not sure of the future viability of those in the areas where I want to live and work. So I think just trying to read what’s going to be ﬁlled by all the huge numbers of doctors coming through that are going to ﬁll specialist positions and then where the role of the GP is actually going and what we still be required to do to ﬁll and where. (I08; Female; PGY3)”* | - Perception of training and working opportunities strongly inﬂuenced career choices among all junior doctors, independently of their specialty and practice location intention. There is only a rural generalist pathway available for doctors who want to stay in rural workplaces, and this frustrate interns who have non-GP intention.   *“I actually ﬁnd it very disappointing after working in rural areas and wanting to go back to those areas so badly, that unless you speciﬁcally want to be that rural GP, there’s ﬁrstly no pathway. And two, it’s not only not encouraged, it’s almost frowned upon. I ﬁnd it amazing because the whole time I was in rural areas people talk about how much they’re trying to bring people rurally. When I look at it I kind ofsee a lot ofclosed doors. (I19; Male; FGY1)”*  *“I think the biggest thing for making people who want to live rurally stay is giving them the opportunity to do their specialty training programs in a rural area. Because the longer you stay somewhere, the more likely you’ll stay. But it doesn’t really help for people to stay in a country area for internship and residency if they can’t be a registrar and stay there long term. (I11; Female; PGY2)”* |
| Harris 2020  UK / FY2 / Specialty choice – GP  CASP 19 (relationship) / Key |  |  |
| Rizan 2019  UK / FY2 / Not going into specialty training  CASP 18 (relationship) / Key | - Taking a F3 as concern over not competitive enough portfolio and the F3 years can build their CV to make them more competitive in the future |  |
| Appleton 2017  UK / Psychiatry trainee / Specialty choice – psychiatry  CASP 20 / Key |  |  |
| Essa 2010  SA / Doctors / General  CASP 17 (recruitment, relationship) / Key |  | - Despite not enjoying working in the public sector, but having the desire to specialize further |
| Croghan 2020  Ireland / Intern / Specialty choice  CASP 16 (relationship, ethics, analysis, finding) / Satisfactory | - Picking a specialty strategically based on growing need, seeing senior doctors encounter a lack of job opportunities and disillusioned by this. However not seem like a strong influence. Interns stating ’at the end of the day I’d mostly just [follow my] interest’ (Richard), or that it would be ‘too difﬁcult to forecast [job openings] anyway, really’ (James). |  |
| Firth 2011  UK / FY2 / Specialty choice – GP  CASP 16 (design, relationship, valuable) / Satisfactory |  |  |
| Liang 2019  Australia and NZ / Women leaving surgical training / others  CASP 19 (statement) / Satisfactory |  |  |
