# Supplementary material for: How do foundation year and internship experience shape doctors’ career intentions and decisions? A meta-ethnography
Source: Med Teach. Author manuscript; Available in PMC 2024 Jan 19. (PMC7615548; doi:10.1080/0142159X.2022.2106839)
Supplement: Appendix 4 [file EMS193419-supplement-Appendix_4.docx]

Appendix 4. CERQual evidence profile

| **Category** | Summary | Assessment of methodological limitations | Assessment of coherence | Assessment of adequacy | Assessment of relevance | Overall CERQual assessment of confidence | Explanation of judgement |
| --- | --- | --- | --- | --- | --- | --- | --- |
| **The “hand-on” experience and “real life” exposure** | Interns may or may not have an intention or interest during medical school and this could be either re-enforced or changed dramatically during internship. The “hand-on”, “real-life” exposure to clinical practice and different specialties make some realize they enjoy it,or in some other cases, they do not enjoy working with certain patients, there are unexpected responsibility with some specialties, or they don’t even want to continue practice medicine, as being a student and being a doctor has significantly different level of responsibility. The comparison between different rotations also helps interns decide which specialty fit them best. | Minor methodological concerns (not clear in a number of studies whether the relationship between the researcher and participants was adequately considered; in another few studies the recruitment and analysis processes were not clearly described) | No or very minor concerns | No or very minor concerns | No or very minor concerns | High confidence | Only minor methodological concerns on relationship between the researcher and participants in a number of studies, and in another few studies the recruitment and analysis processes were not clearly described |
| **Positive experience, confidence and readiness** | After successfully managing patients in different rotations, interns may feel confident and ready to further develop skills in certain specialty and sometimes with supportive consultants and team they rebuild previously damaged confidence; or on the contrary, negative experiences with lack of support could lead to inters feeling uncomfortable, losing confidence and not ready to further continue practicing or enter specialized training. | No or very minor methodological concerns (not clear in a few studies whether the relationship between the researcher and participants was adequately considered) | No or very minor concerns | No or very minor concerns | Moderate concerns (all reported from HIC, no LMIC data) | Moderate confidence | Very minor methodological concerns on relationship between the researcher and participants in a few studies, moderate concerns regarding relevance as all reported from HIC and no LMIC data |
| **The workload, work-life balance, lifestyle** | Interns prefer more manageable workload and healthier work-life balance. During internship, they may experience or observe the actual workload/lifestyle of different specialties, which the actual situation might be different than what they expect (e.g. GPs also work stressful and long hours). Interns will choose careers (specialties, country of work) that fit their preferred lifestyles (some can also sacrifice) and whether it will be compatible with their future personal life of e.g. having a family and children, which also has a gender aspect to it. | Minor methodological concerns (not clear in a number of studies whether the relationship between the researcher and participants was adequately considered; in another few studies the analysis processes were not clearly described) | Minor concerns (all studies reported the importance of work-life balance though one paper also reported it’s less important) | No or very minor concerns | No or very minor concerns | High confidence | Only minor methodological concerns on relationship between the researcher and participants in a number of studies, and in another few studies the analysis processes were not clearly described; and minor concerns regarding coherence. |
| **Well-being, emotional stress and the need to step off** | Internship training is stressful, exhaustive, and sometimes also lonely, especially in certain rotations. Interns feel like they are on a “conveyor belt” or “treadmill” all the time, and will prefer careers that they can bear, and in some situations “step off” and take time out of training to alleviate their stress and exhaustion. | No or very minor methodological concerns (not clear in a few studies whether the relationship between the researcher and participants was adequately considered) | No or very minor concerns | No or very minor concerns | Moderate concerns (all reported from HIC, the one paper from South Africa did not explicitly relate this with career decision/intention) | Moderate confidence | Moderate concerns on relevance as all reported from HIC, the one paper from South Africa did not explicitly relate this with career decision/intention |
| **Image of professions and self-identity** | There are public and social status, respect and characteristics linked with certain specialties that are being reminded to doctors and also during internship. Interns may find this time to reflect and think whether their proposed career fit their self-identity, whether choosing certain specialty will make them being considered as “not a proper doctor”. This is also linked with the hierarchy of career options and specialties. | No or very minor methodological concerns (not clear in a few studies whether the relationship between the researcher and participants was adequately considered) | No or very minor concerns | Serious concerns (3 studies offered thin data and 4 studies offered very thin data) | Serious concerns (all studies reported specialty choices only, not clear if it is directly relevant to internship experience; all studies from HIC, no LMIC data) | Very low confidence | Serious concerns on adequacy as studies only offered thin or very thin data; serious concerns on relevance as all studies reported specialty choices only, not clear if it is directly relevant to internship experience and that all studies from HIC, no LMIC data |
| **Relationship with supervisors/consultants** | Consultants and supervisors are very influential in interns’ career choices. Enthusiastic and supportive supervisors could be mentors, role models, giving career advice (even lobbying) and interns will “want to be like him/her”. In some other cases, there could be insufficient or no role models, or poor ones that bully interns, do not adequately support interns of their practice and career choices, or performing poor clinical practices and interns will “not want to become such doctor”. | Minor methodological concerns (not clear in a number of studies whether the relationship between the researcher and participants was adequately considered; in another few studies the recruitment and analysis processes were not clearly described) | No or very minor concerns | No or very minor concerns | No or very minor concerns | High confidence | Only minor methodological concerns on relationship between the researcher and participants in a number of studies, and in another few studies the recruitment and analysis processes were not clearly described |
| **Relationship with peers** | Peers are important social relationship during internship training that can support each other. Interns make career decisions based on the information sometimes generated from peer networks, and interns may want to make choices that are “validated” by their peers and follow others’ recommendation or what everyone else are doing. Interns may also want to continue working in certain locations or specialties because they want to maintain established personal relationship. However, there are also competition between peers, such competition and “elbowing one’s way into the theatre” may deter interns’ interest into certain career, and some may decide to leave so that there could be less competition for their peers. | Minor methodological concerns (not clear in a number of studies whether the relationship between the researcher and participants was adequately considered; in another few studies the some other aspects were not clearly described) | No or very minor concerns | No or very minor concerns | Minor concerns (most reported from HIC, one LMIC data) | High confidence | Only minor methodological concerns on relationship between the researcher and participants in a number of studies, and in another few studies some other aspects were not clearly described; minor concerns regarding relevance as most but one paper are from HIC |
| **Relationship with senior colleagues and the team** | Other senior colleagues and the healthcare team are also key to career decision-making. Interns prefer teams that are supportive, friendly, approachable, feeling welcomed and valued, and demonstrate similar characteristics – and reject ones that are unsupportive. Interns also could seek advice from other colleagues like specialties trainee to get more familiar with working practices and training requirement, or sometimes just “witnessing registrar that are broken” and therefore shift career plans. | Minor methodological concerns (not clear in a number of studies whether the relationship between the researcher and participants was adequately considered; in another few studies the recruitment, ethics and analysis processes were not clearly described) | No or very minor concerns | No or very minor concerns | No or very minor concerns | High confidence | Only minor methodological concerns on relationship between the researcher and participants in a number of studies, and in another few studies the recruitment, ethics and analysis processes were not clearly described |
| **Relationship with patient and the community** | Through working with patients and the community, interns may realize if they like such interaction, interns may also feel like they are integrated to the community and want to continue serving the community that decided their career choices. | Minor methodological concerns (in a few studies the design, recruitment, ethics and analysis processes were not clearly described) | No or very minor concerns | Minor concerns (3 studies offered moderately rich data, 2 offered thin data and 1 very thin data) | Minor concerns (most reported from HIC, one LMIC data) | Moderate concerns | Minor methodological concerns as in a few studies the design, recruitment, ethics and analysis processes were not clearly described; minor concerns on adequacy as studies offered moderately rich to very thin data; minor concerns regarding relevance as most but one paper are from HIC |
| **Characteristics and hierarchy of career options and specialties** | There are said and unsaid characteristics and hierarchy of different career choices and specialties. Interns constantly hear consultants, senior colleagues or their peers commenting or bashing on some specialties like “just a GP” or “not worthwhile” or some specialties being more gendered e.g. surgery being a male-dominated specialty and females are discouraged from it. Interns sometimes even say this among themselves or have to hide their preferred intention for future career. This inevitably influenced interns’ career decisions. However, not everyone is affected by this (ref 167). | Minor methodological concerns (in a few studies the design, recruitment, ethics and analysis processes were not clearly described) | Minor concerns (all studies reported that the hierarchy impacted their decision-making though one paper also reported quotes that suggest it is not important and they won’t be impacted) | No or very minor concerns | Moderate concerns (all studies reported specialty choices only, not clear if this will be relevant to other career options; most reported from HIC, one LMIC data) | Moderate confidence | Minor methodological concerns as in a few studies the design, recruitment, ethics and analysis processes were not clearly described; minor concerns on coherence; moderate concerns regarding relevance as all studies reported specialty choices only, not clear if this will be relevant to other career options and most reported from HIC, one LMIC data |
| **Workplace location, condition, resources and environment** | Interns are also drawn towards workplaces that have good facilities and resources, supportive environment, and high morale. | Minor methodological concerns (in a few studies the design, recruitment, ethics and analysis processes were not clearly described) | No or very minor concerns | Moderate concern (1 study offered moderately rich data, 1 thin data and 4 very thin data) | No or very minor concerns | Moderate concerns | Minor methodological concerns as in a few studies the design, recruitment, ethics and analysis processes were not clearly described; minor concerns on adequacy as studies offered moderately rich to very thin data |
| **Feeling valued by the organization and healthcare system** | Interns sometimes feeling undervalued and under-appreciated by the organization and the healthcare system. They felt they are considered as “cheap labour” for service provision. They are sometimes frustrated by the administration that lack accountability, does not care about their wellbeing, and consider interns as hassle if they raise concern. Therefore interns may consider choosing other work organizations (private sector, another country) for their future career. | Minor methodological concerns (in a few studies the design, recruitment, ethics and analysis processes were not clearly described) | No or very minor concerns | No or very minor concerns | Minor concerns (most reported from HIC, one LMIC data) | High confidence | Minor methodological concerns as in a few studies the design, recruitment, ethics and analysis processes were not clearly described; minor concerns regarding relevance as most but one paper are from HIC |
| **Job market polices and changes, job security: will I get a job** | The job market polices for example changes to junior doctor contract, government cost-cutting are felt even closer during internship period, this will lead to interns choosing specialties that are more employable in the future or workplaces that have more certainty. However, it’s not always a strong influencer and sometimes it’s hard to predict (ref 144). | Minor methodological concerns (in a few studies the design, recruitment, ethics and analysis processes were not clearly described) | Minor concerns (all studies reported the importance of future job opportunities, though one paper said it’s not strong influence as future is hard to predict) | No or very minor concerns | No or very minor concerns | High confidence | Only minor methodological concerns as in another few studies the recruitment, ethics and analysis processes were not clearly described; and minor concerns on coherence |
| **Future training and professional development opportunities: will I get advanced** | Interns will choose career options that will allow their future training and professional development, based on what they experience with the healthcare team and what they hear about in other settings. Interns won’t choose pathways that limit personal development, or jobs that are low-quality and “no one else want”. | Minor methodological concerns (in a few studies the design, recruitment, ethics and analysis processes were not clearly described) | No or very minor concerns | No or very minor concerns | No or very minor concerns | High confidence | Only minor methodological concerns as in another few studies the recruitment, ethics and analysis processes were not clearly described |
